# Healthcare professionals’ roles, perceptions, and interventions relating to e-cigarette and vaping practices: a scoping review

**DOI:** 10.1186/s12889-026-27153-2

**Published:** 2026-04-01

**Authors:** Nadia Zamri, Stan Earnshaw, Johanna Bou-Samra, Laetitia Hattingh

**Affiliations:** 1 Pharmacy Department, Charleville Hospital, South West Hospital and Health Services, Charleville, QLD Australia; 2 Research Development, Gold Coast Hospital and Health Service, Gold Coast, QLD Australia; 3 School of Pharmacy and Medical Sciences, Griffith University, Gold Coast, QLD Australia; 4 Allied Health, Toowoomba Hospital, Darling Downs Hospital and Health, Toowoomba, QLD Australia; 5 Allied Health Research, Gold Coast Hospital and Health Service, Gold Coast, QLD Australia; 6 School of Pharmacy and Pharmaceutical Sciences, the University of Queensland, Queensland, Australia

**Keywords:** Vaping, E-cigarettes, Electronic cigarettes, Healthcare professionals

## Abstract

**Background:**

Electronic cigarettes (e-cigarettes), or vapes, deliver aerosols containing nicotine and other harmful chemicals. Growing evidence of health risks and international variation in regulatory policies create challenges for healthcare professionals (HPs) in providing patient information, prescribing cessation products, and ensuring safety. This review examines the roles, perceptions, and interventions of HPs regarding e-cigarettes.

**Methods:**

We searched MEDLINE (Ovid), Embase (Elsevier), CINAHL (EBSCOhost), and Web of Science for English-language studies published since 2000 that examined HPs’ knowledge, attitudes, and clinical practices related to vaping. Searches were completed on 3 October 2024. Of 1,070 records identified, 94 met the inclusion criteria. Data were analysed descriptively and synthesized narratively.

**Results:**

Most studies were from the United States (*n* = 48, 51.1%), Australia (*n* = 13, 13.8%), and the United Kingdom (*n* = 12, 12.8%). More consumers were included in studies as participants compared to HPs (71,356 vs. 24,538 participants). Physicians comprised the majority of HPs (*n* = 15,603, 63.6%), followed by mixed clinician groups (*n* = 4,589, 18.7%) and nurses (*n* = 2,585, 10.5%). Most studies reported on HP perceptions (*n* = 74/94, 78.8%) followed by attitudes (*n* = 67/94, 71.3%), knowledge (*n* = 63/94, 67.0%) and clinical practices (*n* = 60/94, 63.8%). Screening practices were reported in 27 studies, with rates ranging from 0% to 75%. Counselling practices were addressed in 29 studies, and prescribing of e-cigarettes for smoking cessation in 34 studies.

**Discussion:**

Research work outside the US, Australia, and the UK is limited. The earliest publications began in 2014, peaking in 2016 and 2022, which may reflect increased e-cigarette use as a smoking cessation tool, access, advertising, and policy changes. Pharmacists and dentists were underrepresented despite their roles in the supply of e-cigarettes and oral health. Perspectives of other allied health professionals and healthcare assistants remain largely unexplored. Screening, counselling, and prescribing e-cigarette practices varied substantially across countries, professions, and patient groups.

**Conclusion:**

HPs’ roles, perceptions, and interventions regarding e-cigarettes differed by country and profession. Further research should include the perceptions and interventions of a broader range of HPs and examine legislative developments and changes in guidelines across different countries, and how these impact clinical practices. These insights are essential for developing evidence-based practice standards to guide HPs in managing e-cigarette use among patients.

**Supplementary Information:**

The online version contains supplementary material available at 10.1186/s12889-026-27153-2.

## Introduction

Electronic cigarettes (EC), also referred to as e-cigarettes or vapes, are devices that deliver aerosols to the user – commonly known as vaping - which may or may not contain nicotine, as well as potentially harmful additives, flavours, and chemicals. According to a 2023 World Health Organisation Report (WHO), 88 countries did not specify a minimum age at which e-cigarettes can be bought, and 74 countries did not have regulations in place to control use, while 35 countries have banned electronic nicotine delivery systems (ENDS) entirely [1, 2]. While e-cigarettes have been included in several guidelines as an aid to smoking cessation, there is low-level evidence of effectiveness at a population level [1, 3]. Conversely, evidence on adverse population health effects is mounting, including increased risk of heart and lung disorders, poisoning, dependence, and negative impacts on brain development leading to learning and anxiety disorders [1, 4, 5]. With available results from only a small number of long-term safety studies in humans, the effects of e-cigarettes are still uncertain [3]. The variation in e-cigarette legislation and the lack of long-term evidence on the safety and risks create unique challenges for healthcare professionals (HPs) in the provision of advice to their patients.

There have been increases in the use of e-cigarettes among younger people in numerous countries, which prompted alerts of vaping as a public health concern. The increased use can be attributed to many reasons, among which are aggressive marketing campaigns, particularly to young people [1, 6, 7] [7]. In Canada, 43.3% of young adults aged 20–24 years and 35.2% of youth aged 15–19 years reported trialling e-cigarettes in 2020 [6]. Factors attributed to the increase in youth vaping include widespread e-cigarette advertising through social media, the availability of attractive flavoured products, social influences, and adolescent brain sensitivity to nicotine leading to addiction [7]. The colourful marketing of the products misleadingly shifts the association of e-cigarettes from a smoking cessation aid, used within a clinical supportive framework, to the illusion of a harmless consumer product that is used for recreational purposes.

Given the increasing evidence of the negative impacts on various aspects of health and international variation in regulatory policies, HPs experience certain challenges, specifically in relation to balanced provision of information, prescribing e-cigarette decisions, and safety considerations. Furthermore, the challenges of developing and enforcing appropriate e-cigarette regulations present unique difficulties in different countries depending on the legal, regulatory, economic, and sociopolitical contexts of each nation [8]. As such, an area of interest is the perspectives and practices of different healthcare practitioners internationally to obtain an understanding and insights into how the changing landscape is impacting the healthcare sector. An understanding of practitioners’ knowledge about e-cigarettes, their attitudes towards the use of e-cigarettes, and how these impact the provision of advice to patients could inform future strategies to address training gaps and practice guidelines.

Systematic and scoping reviews have explored e-cigarettes in relation to the beliefs, attitudes, and practices of specific healthcare professions, for instance, general practitioners [9–13]. However, there is no scoping review at present examining the perception, roles, and interventions by HPs overall, including allied health and dental professionals. Additionally, there is a lack of information comparing HPs’ opinions and practices between different countries and professions. Thus, this scoping review aims to explore HPs’ roles and practices concerning vaping and e-cigarettes to identify potential gaps in knowledge and clinical practices and provide evidence for improving patient care. Specific focus will be given to the actual clinical practices concerning screening, counselling, and prescribing of e-cigarettes to explore the current practices and inform future strategies.

## Methods

This scoping review was conducted following the JBI (previously *Joanna Briggs Institute)* methodology for scoping reviews [14]. The protocol is available on the Open Science Framework (OSF) (https://osf.io/4by35). Reporting adhered to the Preferred Reporting Items for Systematic Reviews and Meta-analyses Extension for Scoping Reviews (PRISMA-ScR) [15].

Scoping review methodology was selected in line with our aims to explore the available evidence on our broad topic of interest, summarise key characteristics, and identify knowledge gaps in existing research [16]. While the methodology did not involve critical appraisals of the quality of included evidence, detailed interpretation of results and informed discussion were completed to provide direction for future research [17].

### Search strategy and sources

An initial search of MEDLINE (Ovid) and Google Scholar was undertaken to identify articles relating to the topic. Keywords from the titles and abstracts, and index terms from relevant articles identified, were used to develop a MEDLINE (Ovid) search strategy, which was then translated to other bibliographic databases.

The following databases were searched for this review: MEDLINE (Ovid), Embase (Elsevier), CINAHL (EBSCO*host*), and Web of Science (Core Collection). Search results were limited to English language studies (due to the authors’ linguistic limitations), published since 2000, with no restriction on publication type. Database searches included terms such as (vaping OR “electronic cigarette*”) AND (“health* professional*”) AND (attitude* OR practice* OR counsel* OR prescri*). The complete search strategies can be accessed in Appendix 1 of the Supplementary Material. The forward citations of all included sources of evidence were screened for additional studies not captured in the database searches. We further screened the first 200 results from a Google Scholar search to identify any publications not retrieved from the other methods.

### Eligibility criteria

This scoping review included primary studies reporting on the knowledge, perceptions, attitudes, clinical practices, and/or barriers to clinical practice of HPs, either directly (e.g., through surveys of HPs) or indirectly (e.g., surveys of consumers on their experiences with HPs), in relation to vaping/e-cigarettes. Studies from any healthcare setting were considered, with no geographic limitations. Studies that focused on healthcare students or smoking cessation clinics were excluded. Smoking cessation clinics were excluded due to the large number of articles found and the uncertainty in the qualifications of the health professionals involved. Conference abstracts were considered for inclusion, provided enough information on the aspects of e-cigarette knowledge, perceptions, attitudes, clinical practices, and/or barriers being investigated was available for extraction. Post‑graduate trainees were deemed eligible for inclusion, as in the United States (US), many trainees function as fully qualified medical professionals. During the screening process, we ensured that all healthcare professionals were fully registered.

### Study selection

**Fig. 1:**
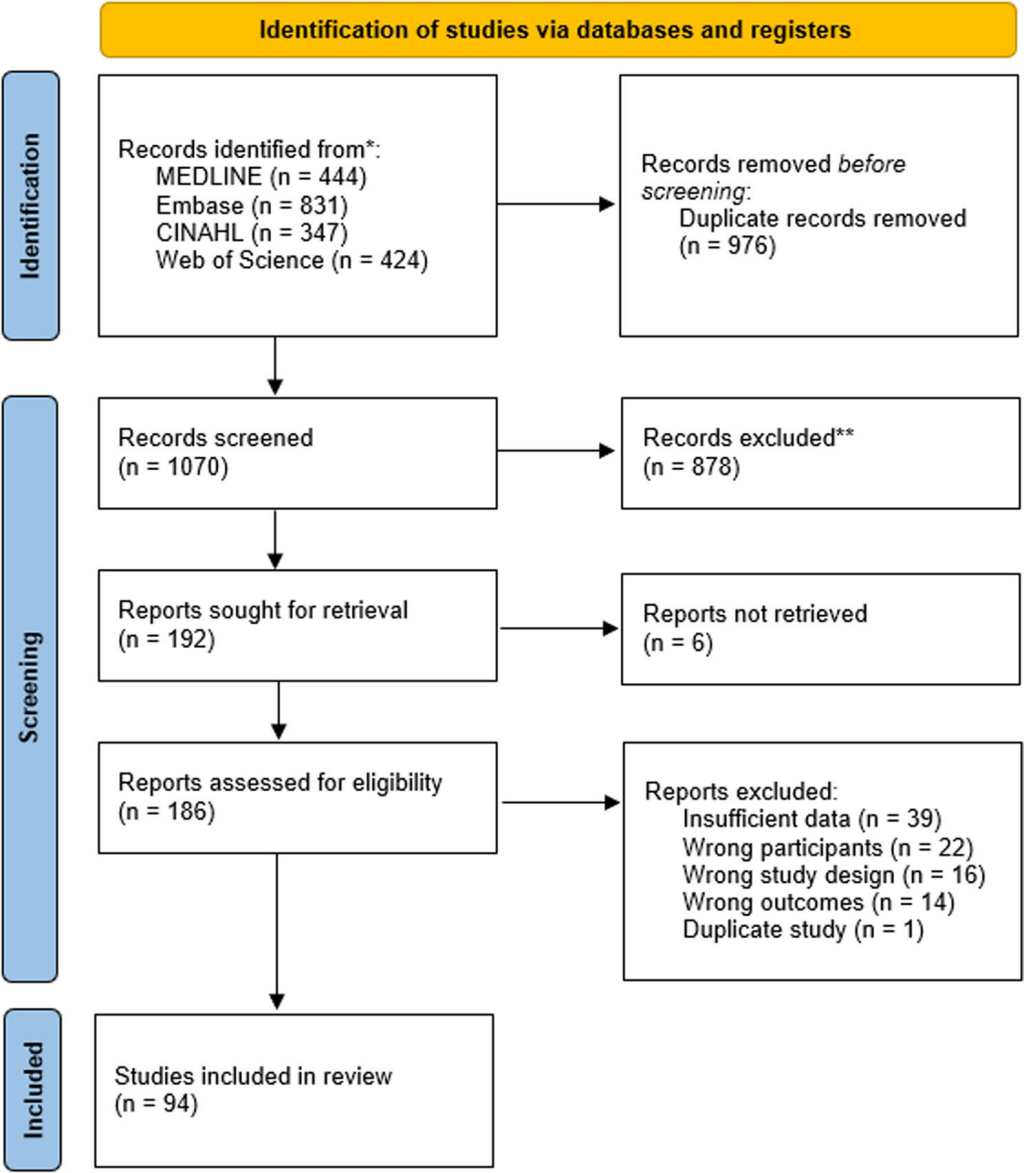
PRISMA flow diagram

The titles and abstracts of records captured by the literature search were first screened against the inclusion criteria independently, by a minimum of two reviewers using Covidence systematic review software (www.covidence.org). The full texts of potentially relevant publications were then assessed in detail against the eligibility criteria by a minimum of two independent reviewers. Reasons for exclusion during full-text screening were recorded and reported. Any disagreements during the selection process were resolved through discussion or by a third reviewer. The results of the search and study selection process have been presented in a PRISMA flow diagram (Fig. 1) [18].

### Data extraction and analysis

In addition to general study and demographic characteristics, we extracted data pertaining to the specific aspects of HPs’ knowledge, perceptions, and practices in the context of vaping and e-cigarettes in the management of their patients, as addressed by the included studies. Extracted data were analysed descriptively in Excel. A summary of all included studies is presented in a table and accompanied by a narrative summary of the results. Areas investigated were also organised into five broad categories: e-cigarette knowledge, perceptions, attitudes, clinical practices, and barriers.

## Results

### Results of the search and selection process

After removal of duplicate records, 1070 articles were identified for title and abstract screening. Subsequently, 186 records underwent full-text screening against the inclusion criteria, of which 94 were included in the review (Fig. 1). Throughout the process, articles were excluded due to unsuitable study designs, wrong participants, insufficient data to be extracted, different data sets focused within the articles, duplication of studies, or dids not focus on e-cigarettes. Detailed characteristics of the 94 included studies can be found in Table 1 of the Supplementary Material.

**Table 1:** Geographic distribution of included studies

Country	No. Studies (%)
United States	48 (51.1)
Australia	13 (13.8)
United Kingdom	12 (12.8)
China	5 (5.3)
Poland	4 (4.3)
Canada	3 (3.2)
England	3 (3.2)
Turkey	3 (3.2)
Ireland	2 (2.1)
Italy	2 (2.1)
Netherlands	2 (2.1)
Slovenia	2 (2.1)
Other	19 (20.2)

### Characteristics of studies

Across the 94 included studies, the majority were conducted in the United States (US) (*n* = 48, 51.1%), followed by Australia (*n* = 13, 13.8%) and the United Kingdom (UK) (*n* = 12, 12.8%). Table 1 details the geographic distribution of the included studies. Five studies [19–23] covered more than one country, and a total of 31 nations were represented across the sample.

Most articles reported on cross-sectional studies (*n* = 90, 95.7%), with the remainder being cohort studies (*n* = 2, 2.13%), quasi-experimental studies (*n* = 1, 1.06%), and randomised controlled trials (*n* = 1, 1.06%). Data was collected primarily through surveys (*n* = 68, 72.3%) followed by interviews (*n* = 21, 22.3%). Two studies (2.1%) combined surveys and interviews [24, 25], and three studies (3.2%) utilised secondary data [26–28]. The collected data were primarily quantitative in nature (*n* = 46, 48.9%), with 20 studies (21.3%) collecting qualitative data, and a further 10 (10.6%) were categorised as mixed methods as they incorporated both quantitative and qualitative data collection approaches.

The research was conducted across a variety of settings, with the majority of studies (*n* = 57, 60.6%) involving a combination of healthcare levels. Table 2 lists the recorded study settings. Mixed settings of various healthcare levels were defined as studies conducted in numerous settings in the community, schools, universities, and/or hospitals.

**Table 2:** Study settings

Study Setting	No. Studies (%)
Mixed settings of various healthcare levels (non-specific settings)	57 (60.6)
Primary care	23 (24.5)
Hospital	12 (12.8)
School (non-tertiary education settings)	1 (1.1)
University	1 (1.1)

**Fig. 2:**
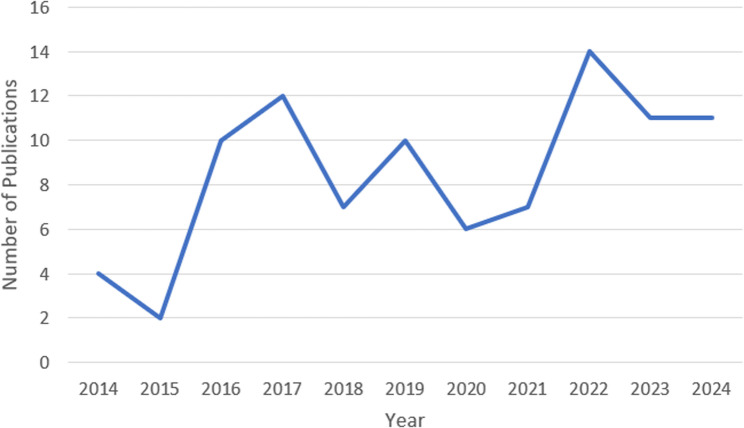
Line graph of the number of publications per year

Since the publication of the earliest included studies in 2014, there has been an upward trend in publications on the knowledge, perceptions, attitudes, and practices of HPs in relation to vaping/e-cigarettes (Fig. 2). Notable spikes in research publications were evident in both 2016 and 2022.

### Study demographics

Medical specialists were the most widely studied HPs (*n* = 39, 41.5%), followed by general practitioners (GPs) (*n* = 36, 38.3%), and nurses (*n* = 31, 33.0%) (Table 3). Consumers, who reported on their experiences with HPs, were involved in 16 (17.0%) of the included studies. A detailed description of study cohorts from each study is provided in Table 1 of the Supplementary Material.

**Table 3:** Participant groups included in studies

Participant Type	No. Studies (%)
Medical Specialists	39 (41.5)
General Practitioners^1^	36 (38.3)
Nurses	31 (33.0)
Physicians (unspecified)	21 (22.3)
Consumers^2^	16 (17.0)
Medical Trainees/Residents	16 (17.0)
Healthcare Professionals (unspecified/other)	14 (14.9)
Pharmacists	9 (9.6)
Allied Health (unspecified/other)	7 (7.4)
Dentists	7 (7.4)
Physician Assistants	7 (7.4)
Psychologists	5 (5.3)
Social Workers	5 (5.3)
Midwives	4 (4.3)
Healthcare Assistants	3 (3.2)
Pharmacy Assistants	1 (1.1)
Physiotherapists	1 (1.1)

^1^General Practitioners includes primary care physicians and family medicine physicians

^2^Consumers are defined as individuals who access health services

The total number of participants across the 94 included studies was 97,023. Consumers were more often studied compared to HPs, with almost three times the number of participants at 71,356 compared to HPs at 24,538. Table 4 provides a breakdown of the number of healthcare workers included across the studies, using simplified categories.

**Table 4:** Number of healthcare professionals included in studies

Healthcare Professional Category	No. Participants (%)
Physicians	15,603 (63.6)
Mixed Clinicians^1^	4,589 (18.7)
Nurses	2,585 (10.5)
Pharmacists	582 (2.4)
Allied Health Clinicians	153 (0.6)
Midwives	139 (0.6)
Dentists	136 (0.6)
Physician Assistants	77 (0.3)
Pharmacy Assistants	76 (0.3)
Healthcare Assistants	37 (0.2)
Other/Unspecified	561 (2.3)
Total	24,538

^1^The category *Mixed Clinicians* was assigned when it was not possible to delineate the composition of HPs (i.e., specific values were not provided), within studies that included multiple clinician types

Physicians made up the vast majority (*n* = 15,603, 63.6%) of the HPs included across the studies, followed by mixed groups of clinicians (*n* = 4,589, 18.7%) and nurses (*n* = 2,585, 10.5%). The remaining HP categories each make up less than 2.5% of study participants.

### Areas of investigation

Areas investigated were organised into five broad categories relating to e-cigarette knowledge, perceptions, attitudes, clinical practices, and barriers.

HP perceptions (i.e., of the benefits and harms of e-cigarettes) were the most widely explored category (*n* = 74, 78.7%). Sixty-three (67.0%) studies examined knowledge of e-cigarettes, 67 (71.3%) studies included the HPs’ attitude, while 60 (63.8%) explored the clinical practices revolving around e-cigarettes. Only 15 (16.0%) studied the barriers in navigating e-cigarettes in terms of screening, counselling, prescribing, and/or recommending e-cigarettes. Table 5 summarizes the specific areas explored across all the studies.

**Table 5:** Areas examined across 94 included studies

Area Examined	No. Studies (%)
Knowledge (*n* = 63, 67%)	
Clinical knowledge/awareness	55 (58.5)
Clinician training	7 (7.4)
Desire for further training	21 (22.3)
Presence/lack of guidelines	8 (8.5)
Perceptions (*n* = 74, 78.7%)	
Benefits of e-cigarettes	55 (58.5)
Harm of e-cigarettes	60 (63.8)
Attitude (*n* = 67, 71.3%)	
Legalization/prohibition	27 (28.7)
Supply/regulation of access	12 (12.8)
Screening for e-cigarette use	5 (5.3)
Counselling on e-cigarettes	24 (25.5)
Clinician Comfort with counselling	34 (36.2)
Prescribing/recommending e-cigarettes	43 (45.7)
Clinical Practice (*n* = 60, 63.8%)	
Screening for e-cigarette use	27 (28.7)
Counselling on e-cigarettes	29 (30.9)
Prescribing/recommending e-cigarettes	34 (36.2)
Barriers (*n* = 15, 16.0%)	
Screening for e-cigarette use	4 (4.3)
Counselling on e-cigarettes	9 (9.6)
Prescribing/recommending e-cigarettes	7 (7.4)

### Clinical practice: screening for e-cigarette use

Of the 94 included studies, 27 discussed HPs’ practices surrounding screening for e-cigarette use. The majority of these studies (*n* = 23, 85.2%) were conducted in the US. Across all populations and settings, the reported proportion of HPs that screen for e-cigarette use ranged from 0% to 75%. While most screening practices were centred around physicians, the screening practices of other professions, including nurses [29–31], pharmacists [32], social workers [29], psychologists [29], dental professionals [33], and physician assistants [29–31], were also explored. Influences for e-cigarette screening included clinician use of e-cigarettes, receipt of training about e-cigarettes, familiarity with tobacco harm reduction (THR), having a standardised process for addressing e-cigarette use, and clinician confidence in their ability to answer patients’ e-cigarette questions [30, 34–36].

Substantial variation in e-cigarette screening practices was noted both within and between countries. In Ireland, one study identified that 31.4% of medical doctors often or always asked about e-cigarettes [37]. A Chinese study found 74.9% of physicians reported that they had never or rarely asked their patients about e-cigarette use [34]. An Australian study reported that 46–50% of GPs and obstetricians often or always asked about e-cigarette use during screening [38]. In the UK, despite awareness of patients utilising e-cigarettes, only 55.1% of pharmacists and 70.3% of physicians incorporated e-cigarette use in assessments [32]. In the US, screening rates of physicians across various specialties ranged between 0% and 75% [26, 30, 39–47]. Few studies compared screening practices across professions. One study noted that nurse practitioners and physician assistants more frequently asked patients about smoking and e-cigarette use compared to oncologists [31].

### E-cigarette screening in special populations

In the context of screening for e-cigarette use in pregnancy, a US study found that only 44% of providers across nurse midwives, nurse practitioners, physician assistants, and physicians attested to asking about ENDS use [48]. Up to 36% of the clinicians noted that they were “unlikely” to incorporate questions about vaping into their standard social history questions. Another US study reported that a third of physicians who provided obstetrical care screened consistently for e-cigarettes/ENDS [49]. An Australian study reported high rates of screening (91.4%) for current e-cigarette use based on the case notes of pregnant women during antenatal care, but observed that screening for lifetime use was significantly lower [28].

From a paediatric perspective, a US study on paediatric healthcare providers noted that they did not screen for e-cigarettes because they were not prepared to discuss them if needed [30]. In another US study, 95% paediatricians similarly did not systematically screen for e-cigarette use [42]. This correlates with two other US-based studies where paediatricians, family physicians, paediatric residents and attending doctors reported asking parents about e-cigarette use significantly less than about cigarette smoking/combustible tobacco use (5%-14% vs. 58%-86%) [44, 50]. Another US study focusing on adolescent healthcare clinicians reported discrepancies between adolescent (10–17 years) and young adult (18–26 years) age groups, with e-cigarette screening rates of 50% and 75%, respectively [43].

### Clinical practice: counselling for e-cigarette use

A total of 29 studies reported on HPs’ e-cigarette counselling practices. The reported proportion of clinicians who provide counselling on e-cigarettes generally ranged between 6.1% and 75.5% [20, 23, 25, 42, 43, 51, 52]. Specific contributing factors reported in studies included clinician confidence in providing counselling [30, 53, 54] and whether the patient or clinician initiated the e-cigarette discussion [35, 47, 48, 55, 56]. Many studies reported patient factors relating to the provision of e-cigarette counselling, including being current tobacco, e-cigarette, or dual users, having made a recent quit attempt, and having depression and/or anxiety [23, 55, 57, 58].

A Polish study focusing on nurses found that 56.8% provided counselling to e-cigarette users as part of lifestyle counselling [59]. A UK study focusing on general practitioners noted that only 33.7% provide advice concerning e-cigarettes during consultations, and only 4.9% refer e-cigarette users to cessation services [51]. A study conducted in Mexico found that 33.7% of adult smokers discussed e-cigarettes with their HPs [55]. The percentage of physicians or primary care providers who counselled concerning e-cigarettes in the US ranged from 15 to 62%, with stark differences between specialties [58, 60, 61]. One study involved four countries in a gradient of ‘most restrictive’ (Australia), ‘somewhat restrictive’ (Canada), and ‘less restrictive’ (England and the US) in relation to the regulation of nicotine vaping products (NVP) [20], a type of e-cigarette product. The study indicated no difference between countries in the prevalence of NVP discussions. However, it was noted that the proportion of HPs initiating NVP discussions increased in England between 2016 and 2020 (53.3–72.8%) but did not significantly change in other countries.

### E-cigarette counselling in special populations

A US study involving obstetrics and gynaecology providers found that the majority (63%) of clinicians who discuss vaping in clinical settings reported that they initiate discussions about vaping with their patients [48]. Concerning paediatrics, studies in the US observed that clinical encounters involving e-cigarettes were rare, and 34–65% of paediatricians had never had discussions [25, 42, 50]. Adolescent healthcare clinicians from another US-based study counselled 20% of their 10–17-year-old and 30% of their 18–26-year-old patients on e-cigarette use [43]. One study reported that paediatric physicians provided advice and quitting assistance to parents who use e-cigarettes at a significantly lower rate compared to combustible tobacco users [44]. Only one study in Australia was identified, which noted that a third of GPs (34%) discussed e-cigarettes with a child or adolescent attending their clinics for other health concerns [52].

### Clinical practice: prescribing/recommending e-cigarette use

Thirty-four studies elaborated on the prescribing of e-cigarette practices of HPs regarding e-cigarettes. Prescribing of e-cigarette practices was defined as what the HPs *were already doing* instead of what they *would do.* Across all populations and settings, the reported proportion of HPs that prescribe or have ever prescribed e-cigarettes ranged from 3.7% to 99.0% [37, 45, 58, 60, 62–68]. For HPs that do recommend/prescribe e-cigarettes, reasons were centred around patients’ tobacco use profiles (current tobacco usage) and cessation attempts, including: as an interim measure to help patients stop smoking completely, as a partial replacement for smoking tobacco, and as a last resort for patients who have failed other treatments for smoking cessation [12, 35, 69–71]. The likelihood of prescribing e-cigarettes was associated with HP knowledge on e-cigarettes, belief in their effectiveness and harm reduction potential, and belief in the sufficiency of evidence in a positive correlation [63, 69, 72]. In terms of HP characteristics, studies reported differences in e-cigarette prescribing by physicians based on gender and specialty, with males and pulmonologists being the most likely prescribers [66], while others report no association [60, 72].

There was substantial variation in the prescribing of e-cigarette practices between and within countries. In Ireland, a study found that 78.1% of medical doctors never recommended e-cigarettes to patients [37]. One study examining family physicians in Turkey reported that 6.6% had recommended ECs to their patients for smoking cessation [72], while another found that 98.8% and 99.0% of physicians had recommended closed and open tank systems of e-cigarettes, respectively [64]. In Mexico, one study found that almost half (46%) of smokers who discussed e-cigarettes indicated that their HP recommended their use, 23.5% reported being advised against their use, and 29.6% indicated their HP did not express an opinion either for or against e-cigarette use [55]. In the US, numerous studies observed prescribing practices, reporting that between 3.7% and 61% of physicians have at some point recommended e-cigarettes to their patients who smoke [36, 45, 46, 58, 60, 62, 63, 66–68].

In a multi-national study [20], it was reported that the proportion of HPs that recommended NVPs was substantially higher in England (55.7%) compared to the US (14.7%), Australia (20.2%), and Canada (25.7%). A similar study between the same four countries found differing results, with the prevalence of HPs recommending NVPs being three times more likely in the US than in Australia, and twice as likely in Canada as in Australia [22].

### E-cigarette prescribing in special populations

An Australian study noted that most GPs (79%) had never recommended e-cigarettes to adolescents who smoked conventional cigarettes, with the remainder only rarely or occasionally making a recommendation [52]. In relation to pregnancy, a US-based study noted that 4.4% of physicians prescribed or recommended e-cigarettes to pregnant smokers as a cessation method [49].

Further details of the results extracted from each study relating to clinical practice are included in Table 3 of the Supplementary Material.

### Barriers to e-cigarette-related practices

Across the studies, there were a number of factors cited as barriers to addressing e-cigarettes in practice. Commonly reported barriers included lack of knowledge, scarce peer-reviewed research, limited time, lack of formal education or training on e-cigarettes, and absence of guidelines or resources [9, 30, 42, 44, 48, 51, 52, 69]. It was further noted that the level of comfort discussing e-cigarettes (both HP and patient), and the therapeutic relationship, play a significant role as a facilitator or barrier to addressing e-cigarettes [39, 52, 69]. Specific to adolescents, HPs reported issues with competing priorities, a lack of appropriate screening or counselling tools, and difficulty in having open conversations about e-cigarettes in the presence of parents [30, 42, 52].

## Discussion

This review aimed to assess HPs’ roles, perceptions, and interventions in relation to vapes and e-cigarettes, especially given the role they play in the context of harm reduction. Most of the 94 studies identified were cross-sectional studies, with data being collected primarily through surveys. The research was conducted across a variety of settings, with most involving a combination of healthcare levels. The areas investigated by this review were broadly categorised into five groups: knowledge, perceptions, attitudes, clinical practices, and barriers.

Vaping, once promoted as a safer alternative to smoking and a cessation aid, has become a significant public health concern—particularly for youth and young adults— as it can lead to addiction and negatively affect respiratory health and brain development [73]. By synthesizing the global evidence, this review identifies gaps in HPs’ knowledge, perceptions, and attitudes that impact their clinical practices, as there are missed opportunities to provide evidence-based information to patients, specifically the youth and young adults. This highlights the importance of equipping HPs with the required tools to address vaping-related health risks.

### Global perspectives and policy landscape

The World Health Organization (WHO) reported that e-cigarettes are addictive and harmful, recommending regulation of these products, which may include strengthening bans on advertising, public use, and flavoured products, to decrease uptake among non-smokers and youth [1, 74]. WHO’s Call to Action further notes that e-cigarettes, irrespective of whether they are commercialised as consumer products for smoking cessation, must involve clinical supervision or advice [74]. However, our study found that there were significant variations, even within single countries, in relation to screening, counselling, and prescribing of e-cigarette practices, which do not align with the protective aims of the WHO. An explanation for the heterogeneity observed across the studies in relation to perceptions, roles, and clinical practices is the regulatory and policy environment in which the HPs practice. Vaping policies range from countries that strictly prohibit use of e-cigarettes (e.g., Turkey, Mexico) [75–77], to medically regulated (e.g., Australia) [78, 79], or harm‑reduction oriented consumer access models (e.g., England) [80, 81]. This review suggests that such policy contexts shape not only what healthcare professionals *can* do, but also what they *professionally incorporate* in their practice to discuss, recommend, or avoid.

Our review also found that most studies were limited to clinicians in the Western context, predominantly in the US, followed by Australia and the UK. Thus, to inform culturally sensitive interventions and observe the existence of clinical practice variations, future research should focus on underrepresented regions, particularly on the low- and middle-income countries [12, 82–84].

### Health professionals’ roles and perceptions

HP perceptions (i.e., on the benefits and harms of e-cigarettes) were the most widely explored category, while a minority of the included studies evaluated barriers to e-cigarette screening, counselling, and prescribing. Only 28.7% of studies assessed HP perspectives on vaping legislation, indicating a need for further research to explore regulatory issues and their implementation in clinical settings. While the literature emphasised that many HPs do not feel adequately equipped to discuss or manage e-cigarette use with patients [85–87], only 7.4% of studies specifically assessed clinician training on e-cigarettes, with 22.3% exploring whether HPs were interested in receiving further training [9, 40, 42, 44, 48, 50, 53, 54, 64, 69, 70, 85]. A better understanding of HP training on e-cigarettes can help to shape educational interventions and resource development, ultimately leading to more consistent, evidence-based practices. This gap is critical, similarly noted in previous literature, which suggests that HPs often do not feel prepared to counsel patients on vaping due to limited knowledge and lack of confidence [87, 88]. The findings highlight the need for structured, evidence-based training programs that address potential therapeutic roles of e-cigarettes. Future studies should also evaluate the effectiveness of such education programs in enhancing clinical practice and patient outcomes.

Since the publication of the earliest included studies in 2014, there has been an ongoing increase in publications on the knowledge, perceptions, attitudes, and practices of HPs in relation to vaping/e-cigarettes, with spikes in research outputs in 2016 and 2022. This could be due to increased e-cigarette and vaping usage over these periods [89], potentially due to a surge in advertising (e.g., multiple flavours), changes in legalisation, perception of e-cigarettes as less harmful, or a rise in awareness of use as a smoking cessation tool [89]. For instance, a study by Ozga et al. [90] reported that e-cigarette advertising expenditures in the US peaked around 2018–2019, with targeted campaigns aimed at youth and young adults. Ozga et al. [90] noted that the marketing efforts likely contributed to heightened public and professional awareness.

Medical specialists were the most widely studied group of HPs, followed by general practitioners (GPs) and nurses. Other professions, such as pharmacists and dentists, were not as widely studied despite their involvement in supply and the relationship between e-cigarettes and oral health. The perspectives of allied health professionals and healthcare assistants have also not been widely captured. This is concerning given the roles these professionals play in tobacco harm reduction, product supply, and oral health monitoring [86]. The lack of inclusion of these professionals may result in missed strategies for comprehensive intervention. As such, future research should explore the unique contributions and training needs of these professions, especially in community and primary care settings where they often serve as first points of patient/consumer contact.

### Clinical practices and barriers

The rates of screening, counselling, and prescribing/recommending e-cigarettes differed substantially across countries, professions, and populations. Interestingly, one study addressed how e-cigarettes may be viewed or navigated in the hospital setting, such as issues around whether vaping should be allowed on hospital grounds [71]. However, no specific study included in this review had explored policies concerning how e-cigarettes should be handled in the hospital setting for inpatients. A limited number of studies focused on special populations such as pregnant women, adolescents, or patients with mental health conditions [25, 28, 30, 42–44, 48–50, 52]. Hence, future studies should explore how to better support HPs in addressing e-cigarette use among high-risk populations and within varied settings.

In the US, the Centers for Disease Control and Prevention (CDC) advises against e-cigarette use among youth, pregnant women, and non-smokers, and emphasises the lack of FDA approval for e-cigarettes as cessation tools [82]. The CDC also elaborates on clinical guidance for managing vaping-related lung injuries, illustrating the need for appropriate screening and counselling of patients by HPs [83]. However, our findings showed that even among targeted patient groups (adolescents and pregnant women), there was stark variation in actual screening and counselling in clinical practice for all the studies across countries and HPs.

Australia represents a distinct policy‑practice tension due to continuously changing policies. In Australia, as of July 2024, all vapes can only be sold in pharmacies, with restrictions on flavours and packaging [84]. These changes reflect a shift toward viewing e-cigarettes as therapeutic tools rather than consumer products, aligning with WHO’s recommendations. While recent policy changes place vaping as a therapeutic product accessible through pharmacies, the review found relatively low rates of prescribing or recommending e‑cigarettes, including among general practitioners. This correlates with findings that a lack of long-term data is a barrier to performing clinical activities in relation to e-cigarettes. This also aligns with a recent mixed-methods systematic review that found GPs have mixed views on recommending e-cigarettes. While some GPs see them as useful cessation tools, others express concerns about safety and lack of long-term evidence [12]. In essence, this may also suggest that regulatory change alone may be insufficient to alter clinical behaviour in the absence of a clear professional consensus. The lag between policy reform and practice uptake also illustrates the importance of implementation supports such as education, prescribing pathways, and medico‑legal clarity.

In the UK, the NICE guidelines (NG209) support the use of e-cigarettes as a smoking cessation aid but note that further research into long-term safety and effectiveness is required [84]. In countries with harm‑reduction–oriented policies, such as England, HPs were more likely to discuss and recommend e-cigarettes as part of smoking cessation. Multi‑country studies demonstrated substantially higher rates of recommendation in England compared with Australia, Canada, and the US [20], aligning with national guidance that positions e‑cigarettes as a supported cessation option. This policy endorsement may have potentially contributed to the normalisation of clinician engagement with vaping, increased professional confidence, and enhanced willingness to initiate discussions with patients.

In contrast, in highly restrictive settings, such as Turkey [75], clinicians reported lower rates of recommending e‑cigarettes and may express neutrality or avoidance in their clinical opinions. However, studies from these contexts suggest that restrictive legislation does not reduce clinical encounters involving vaping. Instead, it may potentially shift discussions into ambiguous territories where HPs feel restrained in providing guidance. This regulatory–practice mismatch may contribute to inconsistent counselling and missed opportunities for harm reduction, particularly among patients who continue to utilise e‑cigarettes even in the presence of bans.

Policy context did not uniformly translate into consistent screening and counselling practices. Even in countries with clearer public health guidance discouraging youth and pregnancy exposure, for example, in the US and Australia [91, 92], screening rates for e‑cigarette use among adolescents and pregnant women remained variable or suboptimal. Across all regulatory settings, the absence of transparent clinical guidance emerged as a critical mediating factor. Only a minority of studies investigated HPs’ understanding of legislation or their confidence navigating legal frameworks, despite over a 25% assessing attitudes toward legalisation or prohibition of e-cigarettes. This gap suggests that many clinicians practice with incomplete or outdated knowledge of vaping policies, limiting the practical impact of regulations on care delivery.

The limited focus on barriers to screening, counselling, and prescribing e-cigarettes may also suggest a disconnect between knowledge and practice, whereby a lack of knowledge of evidence translates to variations in clinical practices. In the studies that had explored the topic, barriers such as lack of training, absence of guidelines, and time constraints were the common threads observed, which highlights the need for evidence-based resources to support clinical decision-making. These barriers may hinder the integration of harm reduction strategies into routine care, particularly for patients who smoke and are considering alternatives. Addressing these would require a multi-level and multi-faceted approach, including institutional support, policy clarity, and integration of e-cigarette education into professional curricula. Future research should investigate how these barriers differ across settings and how they can be mitigated through system-level interventions.

Overall, these findings demonstrate that regulatory and policy environments shape HPs’ perceptions and practices in non‑linear ways. Without clinical guidance, professional training, and system‑level support, reforms may fail to translate into consistent, evidence‑based practice. Understanding this interaction is vital for policymakers and professional bodies working to reduce undesirable variation in care and support HPs in navigating the evolving e-cigarette landscape.

### Limitations of study

Our study has a few limitations, such as the exclusion of certain settings (e.g., smoking cessation clinics) and article types such as conference abstracts, which may have excluded potentially relevant findings. Due to the large number of included studies and the nature of our scoping review, we did not delve into the specifics of each study to provide a comprehensive synthesis of study findings. Studies published in non-English languages or grey literature may offer additional insights not captured in this review.

## Conclusion

This scoping review highlights substantial variation in HPs’ roles, perceptions, and clinical practices relating to e‑cigarettes, affected by professional discipline, patient population, and the regulatory and policy environments in which care is delivered. Although the evidence base has expanded rapidly in recent years, research remains concentrated in Western countries and predominantly among physicians. Hence, there are gaps in understanding the perspectives and practices of pharmacists, dentists, allied health professionals, and healthcare assistants who frequently represent the first points of patient contact.

Across countries, inconsistencies in screening, counselling, and prescribing e-cigarettes indicate that regulatory changes alone may be insufficient to ensure consistent, evidence‑based care. Barriers such as limited training, uncertainty regarding long‑term safety, and lack of profession‑specific guidelines continue to diminish clinicians’ confidence and engagement concerning vaping, particularly for high‑risk populations. Given the evolving regulatory landscape, there is an imminent need to equip HPs with up-to-date knowledge and resources to navigate the complexities of e-cigarette use in clinical settings.

Future efforts should move beyond descriptive assessments of knowledge and attitudes to explore how legislative changes, education, and organisational support influence clinical behaviour. Future endeavours should also prioritise underrepresented professions and regions and investigate systemic barriers to effective intervention. By addressing these gaps, the healthcare systems can better support professionals in delivering consistent, informed care, ultimately contributing to improved public health outcomes in the context of vaping and e-cigarette use.

## Supplementary Information

Supplementary Material 1.

## Data Availability

All data analysed during this study are included in this published article and its supplementary information file.
