# Supplementary material for: Healthcare professionals’ roles, perceptions, and interventions relating to e-cigarette and vaping practices: a scoping review
Source: BMC Public Health. 2026 Apr 1;26:1524. doi: 10.1186/s12889-026-27153-2 (PMC13162525; doi:10.1186/s12889-026-27153-2)
Supplement: Supplementary file 1 — Supplementary Material 1. [file 12889_2026_27153_MOESM1_ESM.pdf]

Supplemental Appendix 1 – Search Strategy

All searches were run on 3 October 2024

MEDLINE (Ovid)

#	Searches
1	Vaping/ or Electronic Nicotine Delivery Systems/
2	(vape* or vaping or electronic cigarette* or e cig* or electronic nicotine delivery system*).tw.
3	1 or 2
4	Attitude of Health Personnel/ or Practice Patterns, Physicians'/ or Practice Patterns, Nurses'/
5	exp Health Personnel/
6	(health* professional* or practitioner* or physician* or nurse* or therapist*).tw.
7	5 or 6
8	Patient Education as Topic/ or Counseling/
9	(role* or view* or belief* or attitude* or perceive or perception* or perspective* or position* or discuss* or recommend* or advi#e or counsel* or practice* or prescri*).tw.
10	8 or 9
11	7 and 10
12	4 or 11
13	3 and 12
14	limit 13 to (english language and yr="2000 -Current")

Embase (Elsevier)

No.	Query
#14	#3 AND #12 AND [english]/lim AND [2000-2024]/py
#13	#3 AND #12
#12	#4 OR #11
#11	#7 AND #10
#10	#8 OR #9
#9	role*:ti,ab OR view*:ti,ab OR belief*:ti,ab OR attitude*:ti,ab OR perceive:ti,ab OR perception*:ti,ab OR perspective*:ti,ab OR position*:ti,ab OR discuss*:ti,ab OR recommend*:ti,ab OR advi?e:ti,ab OR counsel*:ti,ab OR practice*:ti,ab OR prescri*:ti,ab
#8	'patient education'/de OR 'patient counseling'/de

#7	#5 OR #6
#6	'health* professional*':ti,ab OR practitioner*:ti,ab OR physician*:ti,ab OR nurse*:ti,ab OR therapist*:ti,ab
#5	'health care personnel'/exp/mj
#4	'health personnel attitude'/exp OR 'clinical practice'/mj OR 'nursing practice'/mj
#3	#1 OR #2
#2	vape*:ti,ab OR vaping:ti,ab OR 'electronic cigarette*':ti,ab OR 'e cig*':ti,ab OR 'electronic nicotine delivery system*':ti,ab
#1	'vaping'/de OR 'electronic cigarette'/de

CINAHL (EBSCO*host*)

#	Query
S13	S3 AND S12<br>Limiters - Publication Date: 20000101-; English Language
S12	S4 OR S11
S11	S7 AND S10
S10	S8 OR S9
S9	TI (role* OR view* OR belief* OR attitude* OR perceive OR perception* OR perspective* OR position* OR discuss* OR recommend* OR advi?e OR counsel* OR practice* OR prescri*) OR AB (role* OR view* OR belief* OR attitude* OR perceive OR perception* OR perspective* OR position* OR discuss* OR recommend* OR advi?e OR counsel* OR practice* OR prescri*)
S8	(MH "Patient Education") OR (MH "Counseling")
S7	S5 OR S6
S6	TI ("health* professional*" OR practitioner* OR physician* OR nurse* OR therapist*) OR AB ("health* professional*" OR practitioner* OR physician* OR nurse* OR therapist*)
S5	(MH "Health Personnel+")
S4	(MH "Attitude of Health Personnel+") OR (MH "Practice Patterns") OR (MH "Prescribing Patterns")
S3	S1 OR S2
S2	TI (vape* OR vaping* OR "electronic cigarette*" OR "e cig*" OR "electronic nicotine delivery system*") OR AB (vape* OR vaping* OR "electronic cigarette*" OR "e cig*" OR "electronic nicotine delivery system*")
S1	(MH "Vaping") OR (MH "Electronic Cigarettes")

Web of Science (Core Collection)

#	Search Query
1	TS=(vape* OR vaping OR "electronic cigarette*" OR "e cig*" OR "electronic nicotine delivery system*")
2	TS=("health* personnel" OR "health* professional*" OR practitioner* OR physician* OR nurse* OR therapist*)
3	TS=("patient education" OR role* OR view* OR belief* OR attitude* OR perceive OR perception* OR perspective* OR position* OR discuss* OR recommend* OR advi?e OR counsel* OR practice* OR prescri*)
4	#1 AND #2 AND #3 and English (Languages) Timespan: 2000-01-01 to 2024-12-31

Supplemental Table 1 – Characteristics of Included Studies

Study ID	Country	Study Aim/s	Methodology	Data Collection Method	Participants	Total Participants	Setting
Agbonlahor et al., 2023 [1]	United States	Examine the prevalence of HCP advice to adolescents to abstain from using tobacco products (generally) and e-cigarettes (specifically)	Cross-sectional - Quantitative	Survey	14531 students	14531	School
Akhter et al., 2023 [2]	Pakistan	To determine knowledge, awareness, attitude and perception regarding e-cigarette among post graduate medical trainees in Pakistan	Cross-sectional - Quantitative	Survey	406 postgraduate medical trainees	406	Mixed settings of various healthcare levels
Albury et al., 2022 [3]	United Kingdom	To understand primary care clinicians' and patients' reported perceptions about offering and being offered a free e-cigarette for harm reduction in routine practice	Randomized controlled trial - Qualitative	Interview	21 patients, 5 nurses, 3 general practitioners, 2 health-care assistants	32	Primary care
Balaraman et al., 2022 [4]	Malaysia	To identify the smoking cessation provider views towards electronic cigarette (EC) safety and effectiveness as a smoking cessation aid	Cross-sectional - Qualitative	Interview	10 pharmacists, 10 physicians	20	Mixed settings of various healthcare levels
Baldassarri et al., 2017 [5]	United States	To determine chest physician perceptions regarding electronic cigarettes (ECs)	Cross-sectional - Quantitative	Survey	994 mixed clinicians	994	Mixed settings of various healthcare levels
Bascombe et al., 2016 [6]	United States	To explore knowledge, beliefs and clinic-based practices regarding traditional and alternative tobacco products (cigar-like products, smokeless tobacco, hookah, e-cigarettes) and marijuana among rural	Cross-sectional - Qualitative	Interview	15 physicians, 3 nurses, 2 physician assistants	20	Primary care

		and urban Georgia primary healthcare providers					
Bell et al., 2017 [7]	Australia	To explore the attitudes of health practitioners who provide healthcare to people living with HIV (PLHIV) towards tobacco harm reduction (THR)	Cross-sectional - Qualitative	Survey	110 physicians, 51 nurses, 18 other (psychologists, dentists)	179	Mixed settings of various healthcare levels
Boakye et al., 2023 [8]	United States	To study current rates of healthcare provider screening for tobacco use among youth	Cross-sectional - Quantitative	Secondary Data	13434 consumers	13434	Mixed settings of various healthcare levels
Bostan et al., 2022 [9]	Turkey	To determine the knowledge and attitude about new generation tobacco products among physicians	Cross-sectional - Quantitative	Survey	438 medical specialists, 18 family practitioners, 50 physicians (unspecified)	506	Mixed settings of various healthcare levels
Brett et al., 2020 [10]	United Kingdom	Explore UK clinicians' beliefs and behaviours around recommending e-cigarettes as a smoking cessation aid for patients with cancer	Cross-sectional - Quantitative	Survey	202 medical specialists, 201 nurses, 103 general practitioners	506	Mixed settings of various healthcare levels
Broadfield et al., 2023 [11]	United Kingdom	To explore midwives' knowledge of and attitudes to electronic cigarette use in pregnancy, and whether this affected their recommendation for their use in smoking cessation	Cross-sectional - Mixed methods	Survey	122 midwives	122	Mixed settings of various healthcare levels
Brown-Johnson et al., 2016 [12]	United States	Identify patient concerns, provider advice and attitudes, and research needs concerning e-cigarettes	Cross-sectional - Quantitative	Secondary Data	N/A	0	Mixed settings of various healthcare levels
Bruno et al., 2024 [13]	Brazil, United States, India	To investigate medical trainees' knowledge and attitudes toward e-cigarette and hookah use	Cross-sectional - Quantitative	Survey	2036 senior medical students and physicians-in-training	2036	Mixed settings of various healthcare levels

Busher et al., 2024 [14]	Ireland	Assessed the knowledge, attitudes and practices of Irish doctors regarding stop smoking care and electronic cigarettes	Cross-sectional - Quantitative	Survey	143 general practitioners, 49 medical specialists, 58 physicians (unspecified)	250	Mixed settings of various healthcare levels
Caponnetto et al., 2022 [15]	Italy	To investigate the point of view of the health personnel regarding cigarettes, their perception of the importance of these for patients, and the comparison with e-cigarettes	Cross-sectional - Qualitative	Interview	8 psychologists, 5 nurses, 3 psychiatrists, 3 social workers	19	Primary care
Chinwong et al., 2024 [16]	Thailand	(1) Assess healthcare providers' opinions on e-cigarette safety and compare them across professions; (2) Evaluate providers' recommendations for e-cigarettes as a tobacco product cessation tool and identify any associations with their safety perceptions	Cross-sectional - Quantitative	Survey	518 nurses, 173 physicians/dentists, 69 pharmacists	760	Tertiary hospitals
Cho et al., 2022 [17]	Australia, Canada, England, United States	Measure the prevalence and changes in smokers' discussions with health professionals (HPs) about nicotine vaping products (NVPs) and HPs' recommendations about NVPs	Cohort study - Quantitative	Survey	12095 consumers	12095	Mixed settings of various healthcare levels
Clegg et al., 2021 [18]	United Kingdom	To identify enablers and barriers to implementing smoke-free National Health Service (NHS) hospital grounds through a hospital staff survey	Cross-sectional - Mixed methods	Survey	153 nurses, 35 healthcare assistants, 36 allied health professionals (unspecified), 33 physicians	257	Hospital
Craig et al., 2022 [19]	United States	Assess the knowledge of Obstetrics and Gynecology (OBGYN) providers regarding	Cross-sectional - Quantitative	Survey	104 O&G providers (nurses, midwives, physician assistants,	104	Obstetrics and gynaecology

		the safety and usage of ENDS in pregnancy. A secondary objective was to understand providers' practices to increase patient education and awareness of the effects of ENDS on pregnant women and developing fetuses			residents, attending physicians)		
Delnevo et al., 2022 [20]	United States	To examine physician-patient communication regarding e-cigarettes	Cross-sectional - Quantitative	Survey	2058 physicians	2058	Mixed settings of various healthcare levels
Deng et al., 2023 [21]	China	To explore the awareness and perceptions of e-cigarettes among Chinese medical and other groups in the context of the COVID-19 pandemic	Cross-sectional - Quantitative	Survey	502 physicians	502	Mixed settings of various healthcare levels
Doescher et al., 2018 [22]	United States	To explore patient preferences regarding the role of the primary care provider (PCP) in discussing electronic cigarette (e-cigarette) use	Cross-sectional - Quantitative	Survey	568 consumers	568	Mixed settings of various healthcare levels
Dwedar et al., 2019 [23]	Egypt	To assess the level of knowledge and beliefs of electronic cigarette among the health care providers and the general population and to compare the knowledge and the beliefs between them about the electronic cigarette	Cross-sectional - Quantitative	Survey	341 consumers, 252 physicians/nurses	593	Hospital
Egnot et al., 2017 [24]	United States	To evaluate resident physicians' personal use, perceived knowledge and use of electronic cigarettes as a smoking cessation aid for their patients and to determine factors associated with	Cross-sectional - Quantitative	Survey	143 medical residents	142	Hospital

		resident physician stage of adoption of electronic cigarettes in clinical practice					
El-Shahawy et al., 2016 [25]	United States	To explore their beliefs and practices regarding e-cigarette use, and to understand the context in which they might recommend e-cigarette use to their patients who smoke	Cross-sectional - Qualitative	Interview	15 medical specialists/family medicine physicians	15	Primary care
England et al., 2014 [26]	United States	To explore the screening practices and attitudes of obstetricians-gynecologists toward smokeless tobacco products (chewing tobacco, snuff/snus, dissolvable tobacco products, and electronic cigarettes)	Cross-sectional - Quantitative	Survey	252 medical specialists	252	Mixed settings of various healthcare levels
Erku et al., 2019 [27]	Australia	To examine views of pharmacy staff regarding the safety of e-cigarettes compared to nicotine replacement therapies (NRTs) and conventional cigarettes, as well as views on their regulation in Australia	Cross-sectional - Mixed methods	Survey	74 pharmacy assistants, 64 pharmacists	140	Primary care
Feng et al., 2019 [28]	China	To assess the beliefs, attitudes, and confidence in e-cigarette counseling among Chinese physicians and explored the factors related to asking patients about e-cigarette use	Cross-sectional - Quantitative	Survey	353 medical residents, 670 medical specialists	1023	Mixed settings of various healthcare levels
Ferrara et al., 2019 [29]	Bosnia, Croatia, France, Ireland, Italy, Malta, Moldova, Netherlands, Poland, Portugal, Slovenia,	To elicit the current knowledge and perceptions about e-cigarettes and tobacco harm reduction (THR) among medical residents in public health (MRPH)	Cross-sectional - Quantitative	Survey	256 medical residents	256	Mixed settings of various healthcare levels

	Spain, Turkey, United Kingdom						
Gallegos-Carrillo et al., 2020 [30]	Mexico	To describe the prevalence and correlates of adult smokers' discussions about e-cigarettes with their health professionals (HPs) in Mexico, including whether these discussions may lead smokers to use e-cigarettes for smoking cessation	Cross-sectional - Quantitative	Survey	1073 consumers	1073	Mixed settings of various healthcare levels
Geletko et al., 2016 [31]	United States	To compare medical residents and practicing physicians in primary care specialties regarding their knowledge and beliefs about electronic cigarettes (e-cigarettes)	Cross-sectional - Quantitative	Survey	61 medical residents, 54 physicians (unspecified)	114	Mixed settings of various healthcare levels
Gorukanti et al., 2022 [32]	United States	To examine adolescent healthcare clinicians' self-reported screening practices as well as their knowledge, attitudes, comfort level and challenges with screening and counselling adolescents and young adults (AYA) for cigarette, e-cigarette, alcohol, marijuana, hookah and blunt use	Cross-sectional - Quantitative	Survey	771 adolescent healthcare clinicians (medical specialists, family medicine physicians, public health workers, and social workers)	771	Mixed settings of various healthcare levels
Gorzkowski et al., 2016 [33]	United States	To assess pediatricians' knowledge, attitudes, and current clinical practices related to e-cigarettes	Cross-sectional - Qualitative	Interview	37 medical specialists	37	Mixed settings of various healthcare levels
Gould et al., 2017 [34]	Australia	To survey Australian GPs and obstetricians about: (a) their practices of asking pregnant women about their use of e-	Cross-sectional - Quantitative	Survey	199 general practitioners, 178 medical specialists	377	Mixed settings of various healthcare levels

		cigarettes, forms of tobacco use other than regular cigarettes, cannabis use, and exposure to SHS, and (b) to compare differences in the responses of different clinicians' groups					
Gravely et al., 2019 [35]	Australia, Canada, England, United States	The objectives were to examine in four countries: (1) the prevalence of health professionals (HPs) discussions and recommendations to use nicotine vaping products (NVPs); (2) who initiated NVP discussions; (3) the type of HP advice received about NVPs; and (4) smoker's characteristics related to receiving advice about NVPs	Cross-sectional - Quantitative	Survey	6615 consumers	6615	Mixed settings of various healthcare levels
Haber et al., 2014 [36]	United States	To investigate inpatient healthcare providers' knowledge, perceptions, and experience with electronic cigarettes, with the goals of informing educational efforts and guiding policy decisions around hospital-based use of electronic nicotine delivery systems	Cross-sectional - Quantitative	Survey	35 nurses, 33 medical residents, 32 physicians (unspecified), 18 pharmacists, 6 social workers, 18 rehabilitation staff	142	Hospital
Heinly et al., 2023 [37]	United States	To assess pediatrician's knowledge, attitude, and behaviors regarding parental e-cigarette use	Cross-sectional - Quantitative	Survey	50 medical residents, 10 medical specialists	60	Primary care
Huang et al., 2022 [38]	China	To evaluate the belief and perception of electronic cigarettes among medical staff	Cross-sectional - Quantitative	Survey	580 nurses, 448 medical specialists	1028	Hospital

		in the respiratory department of medical institutions located in Fujian Province					
Hunter et al., 2021 [39]	United Kingdom	To explore healthcare professionals' attitudes towards vaping in pregnancy and postpartum; beliefs about the health risks of vaping; perceived barriers and facilitators of vaping in pregnancy; knowledge of current guidelines and policies; and training needs	Cross-sectional - Qualitative	Interview	17 midwives, 15 general practitioners, 10 nurses	42	Mixed settings of various healthcare levels
Hurst et al., 2018 [40]	United States	To explore physicians' attitudes toward and knowledge of electronic cigarettes (or Electronic Nicotine Delivery Systems—ENDS), particularly focusing on personal attitudes held by physicians regarding ENDS use, physician beliefs regarding the relative safety of ENDS, attitudes regarding the efficacy of ENDS as a smoking cessation tool, and how physicians' document ENDS use in the electronic health record (EHR)	Cross-sectional - Qualitative	Interview	17 family medicine physicians	17	Primary care
Hwang et al., 2020 [41]	United States	To systematically explore the perspectives of different health care providers on e-cigarettes and their health implications	Cross-sectional - Mixed methods	Survey and Interview	15 medical residents, 15 medical specialists	30	Mixed settings of various healthcare levels

Isett et al., 2018 [42]	United States	To identify gaps in knowledge that if eliminated could improve surveillance and early detection efforts by dental professionals, potentially preventing long-term use of nicotine products and avoidance of the attendant negative health outcomes of smoking	Cross-sectional - Quantitative	Survey	1722 hygienists/dentists	1722	Mixed settings of various healthcare levels
Jackson et al., 2021 [43]	United Kingdom	To estimate the prevalence and correlates of receipt of GP advice on smoking, what type of advice and support was offered and characteristics and quitting activity associated with different types of advice	Cross-sectional - Quantitative	Interview	7430 consumers	7430	Mixed settings of various healthcare levels
Jankowski et al, 2019 [44]	Poland	To evaluate the knowledge, attitude, and behaviors toward smoking cessation and vaping cessation interventions among physicians in Poland; to identify factors shaping physicians' behaviors toward smoking and vaping cessation interventions; and to assess differences in the tobacco cessation interventions recommended for cigarette smokers and users of electronic cigarettes (e-cigarettes)	Cross-sectional - Quantitative	Survey	423 medical residents/medical specialists	423	Mixed settings of various healthcare levels
Jongebloed et al., 2024 [45]	Australia	To understand how nurses are providing smoking cessation care in general practice	Cross-sectional - Qualitative	Interview	14 nurses	14	Primary care

Kanchustambham et al., 2017 [46]	United States	To determine the attitudes/beliefs, concerns, and practices among physicians at Saint Louis University Hospital (SLU) regarding e-cigarettes	Cross-sectional - Quantitative	Survey	70 medical specialists, 45 medical residents	115	Hospital
Kandra et al., 2014 [47]	United States	To report on a physician survey that measured beliefs, attitudes, and behavior related to e-cigarettes and smoking cessation	Cross-sectional - Quantitative	Survey	128 medical specialists/physicians (unspecified)	128	Mixed settings of various healthcare levels
Khalaf et al., 2023 [48]	China	To evaluate the cognition of healthcare professionals and clinical medical students on nicotine and its replacement therapy in Chengdu	Cross-sectional - Quantitative	Survey	217 healthcare professionals (unspecified)	217	Mixed settings of various healthcare levels
Kollath-Cattano et al., 2016 [49]	United States	To determine characteristics of smokers discussing e-cigarette use with their physician and receiving recommendations from their physician to use e-cigarettes for smoking cessation	Cross-sectional - Quantitative	Survey	2617 consumers	2617	Mixed settings of various healthcare levels
Kollath-Cattano et al., 2019 [50]	United States	To explore physician perceptions and recommendations involving e-cigarettes in the context of smoking cessation counselling, including their opinions about the implementation and content of patient educational materials that focus on e-cigarettes	Cross-sectional - Qualitative	Interview	14 medical specialists	14	Mixed settings of various healthcare levels
Koo et al., 2022 [51]	United States	To identify head and neck cancer (HNC) surgeons' attitudes/perspectives of e-	Cross-sectional - Quantitative	Survey	124 medical specialists, 4 physician assistants, 3 medical residents, 5	136	Mixed settings of various healthcare levels

		cigarette and smoking cessation counseling			healthcare professionals (unspecified)		
Koprivnikar et al., 2020 [52]	Slovenia	To explore how healthcare professionals working in the field of preventive healthcare and smoking cessation in Slovenia communicate with and counsel patients regarding electronic cigarettes and smoking cessation or reduction	Cross-sectional - Quantitative	Survey	356 nurses/midwives, 70 physicians, 52 healthcare professionals (unspecified)	478	Mixed settings of various healthcare levels
Luxton et al., 2018 [53]	Australia	To explore the views and practices of clinicians from diverse disciplines, such as surgeons, anaesthetists, nurses and physiotherapists, in the cardiothoracic perioperative period in Australia	Cross-sectional - Qualitative	Interview	30 medical specialists, 11 nurses, 11 physiotherapists	52	Hospital
Marques et al., 2016 [54]	United Kingdom	To seek community pharmacists' perception on use, safety and possible effectiveness of e-cigarettes as quit smoking tools, and their future regulation	Cross-sectional - Quantitative	Survey	92 pharmacists	92	Primary care
McConaha et al., 2018 [55]	United States	To evaluate pharmacists' and physicians' perception and knowledge of ecigarettes, including comfort level in counselling patients on these products. The study also assessed if, and to what extent, patient e-cigarette usage is collected as a marker of tobacco use status	Cross-sectional - Quantitative	Survey	69 pharmacists, 37 family medicine physicians	106	Primary care

McGee et al., 2021 [56]	United States	To assess knowledge, attitudes, and perceived barriers (KAP) regarding e-cigarette use counselling among adolescent healthcare clinical staff in an urban system, and to compare results between providers and rooming staff	Cross-sectional - Quantitative	Survey	169 clinical staff (physicians, nurses, medical assistants, patient care technicians)	169	Primary care
Mohammad et al., 2023 [57]	Jordan	To determine the prevalence of e-cigs usage among Health Care Professionals (HCPs) in Jordan, as well as to investigate their knowledge and attitudes concerning e-cigs use	Cross-sectional - Quantitative	Survey	250 pharmacists, 209 physicians, 73 dentists	532	Mixed settings of various healthcare levels
Morphett et al., 2024 [58]	Australia	To explore the perspectives of Australian health professionals on Australia's NVP prescription model following regulatory changes in October 2021	Cross-sectional - Qualitative	Interview	9 pharmacists, 7 general practitioners, 2 medical specialists, 21 other (nurses, allied health professionals (unspecified), healthcare professionals (unspecified))	39	Mixed settings of various healthcare levels
Moysidou et al., 2016 [59]	Greece	To evaluate the knowledge and perceptions of Greek healthcare professionals about nicotine, nicotine replacement therapies and electronic cigarettes	Cross-sectional - Quantitative	Survey	136 medical specialists, 77 nurses, 32 dentists, 17 general practitioners	262	Mixed settings of various healthcare levels
Mughal et al., 2018 [60]	United Kingdom	To examine GP perceptions of tobacco and electronic cigarette (EC) products, and their attitudes and behaviours towards product cessation	Cross-sectional - Quantitative	Survey	189 medical residents, 117 general practitioners	306	Primary care

Mungia et al., 2021 [61]	United States	To assess dental practitioner's and community members' knowledge and awareness of e-cigarette use. To develop a dental practitioner–based e-cigarette cessation program	Cross-sectional - Mixed methods	Interview	25 dentists, 23 consumers	48	Mixed settings of various healthcare levels
Mungia et al., 2022 [62]	United States	To test the feasibility of the ReACH Assessment of Knowledge for E-Cigarettes (RAKE) cessation program targeting youth and young adult in Texas	Quasi-experimental - Quantitative	Survey	12 consumers, 6 dentists, 1 hygienist	19	Mixed settings of various healthcare levels
Naylor et al., 2024 [63]	New Zealand	To explore the subjective experiences of consumers and staff members regarding the availability of electronic nicotine delivery systems (ENDS) in inpatient mental health units	Cross-sectional - Qualitative	Interview	7 consumers, 9 mental health staff (nurses, healthcare professionals (unspecified))	16	Hospital
Nickels et al., 2017 [64]	United States	To assess physician knowledge/beliefs, self-efficacy, and experience/practice patterns surrounding smoking cessation and electronic cigarettes	Cross-sectional - Quantitative	Survey	464 medical specialists, 97 family practice physicians	561	Mixed settings of various healthcare levels
Northrup et al., 2017 [65]	United States	To assess perceptions of prevalence, safety, and screening practices for cigarettes and secondhand smoke exposure (SHSe), marijuana (and synthetic marijuana), electronic nicotine delivery systems (ENDS; eg, e-cigarettes), nicotine-replacement therapy (NRT),	Cross-sectional - Quantitative	Survey	417 family medicine physicians	417	Primary care

		and smoking-cessation medications during pregnancy, among primary care physicians (PCPs) providing obstetric care					
Ofei-Dodoo et al., 2017 [66]	United States	To explore family physicians' perceptions of recommending e-cigarettes as smoking cessation aids to patients who smoke cigarettes	Cross-sectional - Mixed methods	Survey	55 medical residents, 27 family physicians, 25 physicians (unspecified)	107	Hospital
Ofei-Dodoo et al., 2020 [67]	United States	To evaluate if the 2019 outbreak of EVALI and resultant medical and media reports were associated with a change in family physicians' perceptions of e-cigarettes as tobacco use cessation tools	Cross-sectional - Mixed methods	Survey	98 family physicians, 91 medical residents, 56 physicians (unspecified)	245	Mixed settings of various healthcare levels
Oliver et al., 2022 [68]	United States	To describe the development, implementation, and evaluation of the program's impact on participants' knowledge, attitudes, and practices regarding treatment of adolescent vaping from registration to the end of the series	Cohort study - Quantitative	Survey	72 social workers, 59 nurses, 22 primary care physicians, 2 medical specialists, 2 physician assistants, 2 psychologists, 251 healthcare professionals (unspecified)	410	Mixed settings of various healthcare levels
Pepper et al., 2015 [69]	United States	To understand how physicians communicate about e-cigarettes when counseling adolescent patients and their parents as well as explore physicians' support for regulations aimed at discouraging adolescents' e-cigarette use	Cross-sectional - Quantitative	Survey	410 medical specialists, 366 family medicine physicians	776	Primary care

Pepper et al., 2014 [70]	United States	To describe healthcare providers' awareness of e-cigarettes and to assess their comfort with and attitudes toward discussing e-cigarettes with adolescent patients and their parents	Cross-sectional - Quantitative	Survey	258 family medicine physicians, 189 nurses, 114 medical specialists	561	Mixed settings of various healthcare levels
Peterson et al., 2018 [71]	United States	To investigate pediatric primary healthcare providers' (PPCPs) perceptions about e-cigarettes and current e-cigarette-related clinical practices with adolescents	Cross-sectional - Qualitative	Interview	11 family care physicians, 9 medical specialists, 3 physician assistants, 2 nurses	25	Primary care
Rahman et al., 2024 [72]	Australia	To explore if healthcare professionals felt equipped to support young people to quit vaping	Cross-sectional - Qualitative	Interview	3 general practitioners, 2 psychologists, 1 medical specialist, 1 nurse, 1 pharmacist, 4 healthcare workers (unspecified)	12	Mixed settings of various healthcare levels
Ridner et al., 2017 [73]	United States	To explore the knowledge and attitudes of primary care nurse practitioners (NPs) toward e-cigarettes	Cross-sectional - Quantitative	Survey	93 nurses	93	Primary care
Salloum et al., 2021 [74]	United States	To identify factors influencing primary care physician (PCP) recommendation of e-cigarettes for smoking cessation	Cross-sectional - Quantitative	Survey	103 medical specialists, 98 family medicine physicians, 15 physicians (unspecified)	216	Primary care
Selamoglu et al., 2024 [75]	Australia	To explore general practitioners (GPs) perceptions about the role of e-cigarettes, and understand factors informing their intentions to prescribe e-cigarettes as part of a smoking cessation plan	Cross-sectional - Qualitative	Interview	13 general practitioners	13	Primary care

Sherratt et al., 2016 [76]	United Kingdom	To explore e-cigarette use among patients and examine current practice among clinicians	Cross-sectional - Quantitative	Survey	78 medical specialists, 52 nurses, 1 allied health professional, 1 healthcare professional (unspecified)	132	Mixed settings of various healthcare levels
Shin et al., 2017 [77]	Korea	To explore the views of lung cancer specialists regarding smoking-related policies in Korea	Cross-sectional - Quantitative	Survey	185 medical specialists	185	Hospital
Simoneau et al., 2017 [78]	United States	To understand smoking cessation attitudes of parents and the behaviors, confidence and self-efficacy of pediatricians related to providing smoking cessation counseling to parents and youth	Cross-sectional - Mixed methods	Survey and Interview	1549 consumers, 95 medical specialists	1644	Primary care
Singh et al., 2017 [79]	United States	To explore the knowledge, beliefs, communication, and recommendation of e-cigarettes among physicians of various specialties	Cross-sectional - Qualitative	Interview	25 medical specialists, 10 primary care physicians	35	Mixed settings of various healthcare levels
Singh et al., 2024 [80]	Australia	To explore the Knowledge, Attitude, and Practice of General Practitioners (GPs) in Sydney regarding the use of e-cigarettes in children and adolescents and identify the barriers for GPs to managing children and adolescents using e-cigarettes	Cross-sectional - Quantitative	Survey	53 general practitioners	53	Primary care
Smith et al., 2019 [81]	United Kingdom	To qualitatively explore how mental health professionals' (MHPs) perceive tobacco harm reduction (THR) and electronic cigarettes (ECs), and how these influence their decisions	Cross-sectional - Qualitative	Interview	5 nurses, 5 psychologists, 4 medical specialists, 4 social workers, 2 allied health professionals, 11 healthcare professionals (unspecified)	31	Mixed settings of various healthcare levels

		regarding their use in clinical settings					
Steinberg et al., 2015 [82]	United States	To investigate physician–patient communication regarding e-cigarettes	Cross-sectional - Quantitative	Survey	96 medical specialists, 62 primary care physicians	158	Mixed settings of various healthcare levels
Stepney et al., 2019 [83]	United Kingdom	To explore practitioners' perceptions and attitudes towards e-cigarettes, and their experiences of discussing e-cigarettes with patients	Cross-sectional - Qualitative	Interview	15 general practitioners, 8 nurses	23	Mixed settings of various healthcare levels
Stevens et al., 2024 [84]	Australia	To explore contemporaneous practices around screening and interventions for substance use among pregnant women during routine antenatal care	Cross-sectional - Quantitative	Secondary Data	N/A	0	Hospital
Talley et al., 2017 [85]	United States	To understand the knowledge and perceptions related to electronic nicotine delivery systems (ENDS) among healthcare providers (HCP) practising in the family and the general practice settings	Cross-sectional - Mixed methods	Survey	48 general practitioners, 24 nurses, 6 physician assistants, 2 healthcare professionals (unspecified)	80	Primary care
Tanriover et al., 2022 [86]	Turkey	Determine family physician's level of awareness and harm reduction perceptions of electronic cigarettes	Cross-sectional - Quantitative	Survey	271 family physicians	271	Primary care
Tildy et al., 2023 [87]	Australia, Canada, England, United States	Assess whether health professional interactions regarding smoking cessation and nicotine vaping products differ by mental health condition	Cross-sectional - Quantitative	Survey	11040 consumers	11040	Mixed settings of various healthcare levels
Van Gucht et al., 2016 [88]	Belgium	To compare two groups of healthcare providers in Flanders with regard to their	Cross-sectional - Quantitative	Survey	54 general practitioners	54	Mixed settings of various healthcare levels

		attitudes and risk perceptions concerning e-cigs, registered tobacco counselors having obtained a training and being certified as "tabacologists" and general practitioners (GPs)					
Westmaas et al., 2023 [89]	United States	To investigate the extent to which the cessation practices of oncologists, primary care physicians (PCPs), nurse practitioners (NPs), and physician assistant (PAs) are associated with variables identified in previous research as being associated with (or hypothesized to be associated with) the provision of cessation treatment in clinical settings	Cross-sectional - Quantitative	Survey	151 medical specialists, 150 primary care physicians, 98 nurses, 60 physician assistants	459	Mixed settings of various healthcare levels
Zgliczynski et al., 2019 [90]	Poland	Assess the knowledge and beliefs about e-cigarettes among physicians in Poland	Cross-sectional - Quantitative	Survey	412 physicians	412	University
Zhong et al., 2023 [91]	China	To assess the knowledge and awareness of nicotine, nicotine replacement therapy (NRT), and electronic cigarettes (e-cigarettes) among general practitioners with a special interest (GPwSIs) in respiratory medicine	Cross-sectional - Quantitative	Survey	102 general practitioners	102	Mixed settings of various healthcare levels
Zhou et al., 2020 [92]	United States	Assess the comfort level and practice patterns of physicians with regards to electronic cigarettes to identify gaps	Cross-sectional - Quantitative	Survey	222 primary care physicians, 69 medical specialists	291	Mixed settings of various healthcare levels
Zijlstra et al., 2022 [93]	Netherlands	To obtain an overview of the knowledge and viewpoints on the effectiveness and use of	Cross-sectional -	Survey	59 nurses, 37 general practitioners	96	Mixed settings of various healthcare levels

		smoking cessation interventions (SCIs)	Mixed methods				
Znyk et al., 2024 [94]	Poland	To examine the correlates of counseling provided by primary care nurses with the health status/health behaviors of nurses and the barriers in the advice provided	Cross-sectional - Quantitative	Survey	331 nurses	331	Primary care

Supplemental Table 2 – Data Categories

Study ID	Knowledge				Perception		Attitude						Clinical Practice			Barriers		
	Clinical knowledge	Clinician training	Desire for further training	Presence/lack of guidelines	Benefits of e-cigarettes	Harms of e-cigarettes	Legalisation/ prohibition	Supply/regulation of access	Screening	Counselling	Comfort with counselling	Prescribing/ recommending	Screening	Counselling	Prescribing/ recommending	Screening	Counselling	Prescribing/ recommending
Agbonlahor et al., 2023 [1]														✓				
Akhter et al., 2023 [2]	✓		✓		✓	✓	✓		✓	✓	✓	✓						
Albury et al., 2022 [3]					✓	✓				✓	✓	✓						
Balaraman et al., 2022 [4]	✓				✓	✓	✓				✓	✓		✓	✓			
Baldassarri et al., 2017 [5]					✓	✓				✓	✓	✓	✓					
Bascombe et al., 2016 [6]	✓		✓		✓	✓				✓	✓	✓	✓	✓	✓			
Bell et al., 2017 [7]		✓			✓	✓												
Boakye et al., 2023 [8]													✓					
Bostan et al., 2022 [9]	✓		✓		✓	✓	✓				✓	✓			✓			
Brett et al., 2020 [10]	✓		✓		✓	✓	✓				✓	✓			✓			
Broadfield et al., 2023 [11]	✓				✓	✓						✓						

Brown-Johnson et al., 2016 [12]					✓	✓	✓											
Bruno et al., 2024 [13]	✓				✓	✓					✓	✓						
Busher et al., 2024 [14]						✓							✓		✓			
Caponnetto et al., 2022 [15]					✓													
Chinwong et al., 2024 [16]						✓						✓						
Cho et al., 2022 [17]														✓	✓			
Clegg et al., 2021 [18]						✓	✓				✓							
Craig et al., 2022 [19]	✓	✓	✓			✓			✓	✓	✓	✓	✓	✓				
Delnevo et al., 2022 [20]				✓	✓	✓						✓			✓			
Deng et al., 2023 [21]	✓				✓	✓	✓											
Doescher et al., 2018 [22]	✓													✓				
Dwedar et al., 2019 [23]	✓				✓	✓												
Egnot et al., 2017 [24]	✓					✓						✓			✓			
El-Shahawy et al., 2016 [25]	✓				✓	✓				✓		✓	✓	✓	✓			
England et al., 2014 [26]			✓			✓							✓					

Erku et al., 2019 [27]	✓		✓	✓	✓	✓	✓	✓		✓					✓			
Feng et al., 2019 [28]		✓			✓	✓				✓	✓	✓	✓					
Ferrara et al., 2019 [29]	✓				✓	✓						✓						
Gallegos-Carrillo et al., 2020 [30]														✓	✓			
Geletko et al., 2016 [31]	✓				✓	✓												
Gorukanti et al., 2022 [32]	✓		✓		✓	✓				✓	✓	✓	✓	✓		✓	✓	
Gorzkowski et al., 2016 [33]	✓				✓	✓					✓		✓	✓		✓	✓	
Gould et al., 2017 [34]													✓					
Gravely et al., 2019 [35]														✓	✓			
Haber et al., 2014 [36]	✓				✓		✓											
Heinly et al., 2023 [37]	✓	✓	✓								✓		✓	✓			✓	
Huang et al., 2022 [38]					✓	✓												
Hunter et al., 2021 [39]	✓	✓	✓		✓	✓	✓			✓	✓	✓					✓	
Hurst et al., 2018 [40]					✓	✓	✓		✓			✓	✓	✓				
Hwang et al., 2020 [41]	✓					✓												

Isett et al., 2018 [42]	✓					✓							✓					
Jackson et al., 2021 [43]															✓			
Jankowski et al, 2019 [44]	✓												✓	✓				
Jongebloed et al., 2024 [45]					✓						✓						✓	
Kanchustambham et al., 2017 [46]	✓				✓	✓	✓							✓	✓			
Kandra et al., 2014 [47]	✓				✓	✓							✓		✓			
Khalaf et al., 2023 [48]	✓					✓	✓	✓				✓						
Kollath-Cattano et al., 2016 [49]														✓	✓			
Kollath-Cattano et al., 2019 [50]					✓	✓				✓		✓	✓	✓	✓			
Koo et al., 2022 [51]					✓	✓						✓	✓		✓			
Koprivnikar et al., 2020 [52]	✓			✓						✓		✓			✓			
Luxton et al., 2018 [53]	✓				✓	✓				✓	✓							
Marques et al., 2016 [54]			✓		✓	✓		✓										
McConaha et al., 2018 [55]	✓				✓	✓					✓		✓					
McGee et al., 2021 [56]	✓		✓			✓				✓	✓						✓	

Mohammad et al., 2023 [57]	✓	✓			✓	✓	✓				✓				✓			
Morphett et al., 2024 [58]	✓					✓	✓	✓				✓					✓	✓
Moysidou et al., 2016 [59]	✓				✓	✓	✓	✓				✓			✓			
Mughal et al., 2018 [60]						✓								✓				
Mungia et al., 2021 [61]	✓		✓							✓	✓	✓						
Mungia et al., 2022 [62]	✓		✓		✓	✓				✓	✓	✓						
Naylor et al., 2024 [63]				✓	✓	✓	✓	✓										✓
Nickels et al., 2017 [64]	✓				✓	✓					✓	✓	✓		✓			
Northrup et al., 2017 [65]	✓					✓							✓		✓			
Ofei-Dodoo et al., 2017 [66]					✓	✓	✓					✓			✓			✓
Ofei-Dodoo et al., 2020 [67]					✓	✓	✓					✓			✓			✓
Oliver et al., 2022 [68]	✓					✓				✓	✓		✓					
Pepper et al., 2015 [69]			✓			✓	✓	✓				✓	✓	✓				
Pepper et al., 2014 [70]	✓		✓			✓				✓	✓							
Peterson et al., 2018 [71]	✓					✓	✓	✓	✓	✓	✓		✓	✓		✓	✓	

Rahman et al., 2024 [72]	✓		✓	✓			✓	✓	✓			✓				✓		✓
Ridner et al., 2017 [73]					✓	✓		✓										
Salloum et al., 2021 [74]	✓			✓	✓	✓				✓	✓		✓		✓			
Selamoglu et al., 2024 [75]	✓		✓		✓	✓					✓	✓			✓			✓
Sherratt et al., 2016 [76]	✓		✓	✓		✓					✓			✓	✓			
Shin et al., 2017 [77]	✓				✓	✓	✓	✓		✓	✓	✓						
Simoneau et al., 2017 [78]						✓								✓				
Singh et al., 2017 [79]	✓				✓	✓						✓		✓	✓			
Singh et al., 2024 [80]	✓				✓	✓					✓	✓		✓	✓		✓	
Smith et al., 2019 [81]	✓				✓	✓						✓			✓			✓
Steinberg et al., 2015 [82]															✓			
Stepney et al., 2019 [83]			✓	✓	✓	✓				✓	✓	✓		✓				
Stevens et al., 2024 [84]												✓	✓					
Talley et al., 2017 [85]	✓				✓	✓		✓										
Tanriover et al., 2022 [86]											✓				✓			

Tildy et al., 2023 [87]														✓	✓			
Van Gucht et al., 2016 [88]	✓				✓	✓	✓			✓		✓		✓				
Westmaas et al., 2023 [89]	✓												✓	✓				
Zgliczynski et al., 2019 [90]	✓	✓	✓		✓	✓	✓											
Zhong et al., 2023 [91]	✓				✓	✓						✓						
Zhou et al., 2020 [92]	✓				✓	✓					✓	✓	✓					
Zijlstra et al., 2022 [93]					✓					✓		✓						
Znyk et al., 2024 [94]														✓				

Supplemental Table 3 – Clinical Practices

Study ID	Study Findings
**Screening**	
Baldassarri et al., 2017 [5]	Less than half (44%) reported asking patients about e-cigarette use either most of the time or always.
Bascombe et al., 2016 [6]	Many participants mentioned that, in general, e-cigarette use does not come up in clinic visits very often; however, usually when it was mentioned, the conversation was about using them to quit smoking traditional cigarettes.
Boakye et al., 2023 [8]	31.5% of 13,434 clients was screened for e-cigarette use specifically.
Busher et al., 2024 [14]	31.4% of doctors often or always asked about e-cigarettes.
Craig et al., 2022 [19]	Only 44% (46/104) of OBGYN providers routinely inquired about electronic nicotine delivery systems (ENDS) use during prenatal visits.
El-Shahawy et al., 2016 [25]	When asked about their screening for tobacco use, primary care physicians reported no such process for addressing non-combustible tobacco products.
England et al., 2014 [26]	Of 252 total eligible respondents (those currently providing obstetrics care) 53% reported screening pregnant women at intake for non-combustible tobacco product use all or some of the time, and 40% reported none of the time.<br><br>40% of respondents reported that they never ask patients at intake about their non-combustible tobacco use; 29.0% of the respondents reported that they ask sometimes, 24.2% of them reported that they always ask; and 6.8% of the respondents did not answer the question.
Feng et al., 2019 [28]	A high frequency of asking patients about e-cigarette use is significantly associated with physicians having used e-cigarettes (OR = 2.05), having received training about e-cigarettes (OR = 3.13), and being confident about their ability to answer patients' questions about e-cigarettes (OR = 2.45). Showing a more positive attitude toward using e-cigarettes to quit (OR = 0.79) significantly decreases the odds of high frequency of asking patients about e-cigarette use.<br><br>Gender, age, specialty, smoking status, and risk beliefs of e-cigarettes are not associated with the frequency of Chinese physicians asking their patients about e-cigarette use.

Gorukanti et al., 2022 [32]	10–17 year-old patients: clinicians screened 50.0% of patients for e-cigarette use. 18–26 year-old patients: clinicians screened 75.0% of patients for e-cigarette use.
Gorzkowski et al., 2016 [33]	Most (95%) paediatricians did not systematically screen for e-cigarette use, noting that existing screeners were not comprehensive, and meaningful use criteria ask only about tobacco or cigarettes.
Gould et al., 2017 [34]	13–14% of general practitioners and obstetricians asked “often-always” about e-cigarettes.
Heinly et al., 2023 [37]	Participants reported “asking” parents about e-cigarette use significantly less than about combustible tobacco use (5% vs 58%, P <.001).
Hurst et al., 2018 [40]	Screening for e-cigarette use: Nearly all the physicians reported that they rarely screen for the use of Electronic Nicotine Delivery Systems (ENDS), even with documented smokers they are counselling on tobacco cessation.<br><br>Documenting e-cigarette use: Many physicians reported documenting e-cigarette use in electronic health records. One physician expressed apprehension about documenting ENDS in a formal manner as it may create a disadvantage for them e.g. with life insurance. Another physician noted that they will not be planning to do anything documentation of use until guidelines or regulatory agencies enforce it. One physician stated that unless he engaged in what was considered “extensive discussion” with a patient about smoking, it is unlikely he would even think to probe about ENDS use and would not feel compelled to make any notations in the patient’s electronic health records.
Isett et al., 2018 [42]	The survey results show that a considerable percentage of dental professionals do not screen for e-cigarettes (38%).
Jankowski et al, 2019 [44]	46.8% of the 330 physicians never asked a patient about smoking cigarettes or e-cigarette use when collecting anamnesis.<br><br>Physicians who declared hospital as place of primary employment, compared to those in ambulatory care, were more likely (OR = 3.6; p < 0.01) to ask patients about smoking cigarettes when routinely collecting anamnesis. Physicians with more years of professional experience were more likely to ask a patient about e-cigarette use (OR = 1.1; p < 0.05).<br><br>Females (OR = 2.7; p < 0.05) as well as those who had ever (OR = 9.5; p < 0.05) and never tried e-cigarettes (OR = 12.2; p < 0.05) were more likely to write down in the medical documentation the patients’ cigarette smoking status comparing to males and current e-cigarette users.
Kandra et al., 2014 [47]	48.4% of physicians responding that patients ask about e-cigarettes frequently or sometimes. Only 20.5% of physicians indicated they are never asked about e cigarettes.

Kollath-Cattano et al., 2019 [50]	Physicians did not routinely assess e-cigarette use among patients and reported that discussions were often initiated by patients.
Koo et al., 2022 [51]	Seventy-three (53.68%) of surveyed head and neck surgeons regularly inquire about e-cigarette use. Those familiar with tobacco harm reduction (THR) (63.79%) routinely inquire about e-cigarette use, compared to those unfamiliar with THR (46.15%), with statistical significance between groups ($p = 0.0496$).
McConaha et al., 2016 [55]	Despite awareness of patients utilising e-cigarettes, only 38 pharmacists (55.1%) and 26 physicians (70.3%) included these products in their assessment of patient tobacco use.
Nickels et al., 2017 [64]	58% of family physicians report that they ask their patients about e-cigarette use at least some of the time.
Northrup et al., 2017 [65]	Around a third of the sampled physicians screened for e-cigarettes/ Electronic Nicotine Delivery Systems (33%).
Oliver et al., 2022 [68]	Pre-test data suggested that formal screening about vaping and tobacco was the least common, while asking adolescent patients about tobacco/cigarette use was the most common. Post-test data indicated significant increases in frequency of all screening approaches except in asking parents/caregivers about adolescent patients' vaping and cigarette use.
Pepper et al., 2015 [69]	Significantly fewer physicians routinely screened for e-cigarette use than for cigarette smoking (14% vs. 86% often or always screened; $p < .001$)
Peterson et al., 2018 [71]	Some providers explained that they did not screen for e-cigarettes because they wouldn't be prepared to further discuss it if needed.
Salloum et al., 2021 [74]	About half of primary care physicians reported they often or always ask patients about e-cigarette use.
Stevens et al., 2024 [84]	Across all substances, significantly higher rates of screening occurred for current use versus past use ($p < 0.001$ in all cases). Within both timeframes, women were most likely to be screened for tobacco and least likely to be screened for e-cigarette use based on the review of antenatal care records.
Westmaas et al., 2023 [89]	Nurse practitioners and physician assistants more frequently asked patients about smoking and e-cigarette use compared to oncologists.
**Counselling**	
Agbonlahor et al., 2023 [1]	Among adolescents screened for tobacco use, 76.19% received health care providers' (HCPs) advice to abstain from using tobacco and 75.49% received advice to abstain from e-cigarettes. Among adolescents who did not receive tobacco screening (i.e., were not asked about tobacco products), 68.18% did not receive HCP advice to abstain from

	tobacco products and 64.05% did not receive advice to abstain from e-cigarettes. Among adolescents who were screened for e-cigarette use (32.00% of total sample), 56.65% received HCP advice to abstain from using tobacco products and 70.33% received advice to abstain from using e-cigarettes. Of adolescents not screened for e-cigarette use (68.00% of total sample), 82.62% did not receive HCP advice to abstain from tobacco products and 85.48% did not receive advice to abstain from e-cigarettes.
Balaraman et al., 2022 [4]	Physicians were more likely to recommend e-cigarettes when their patients asked about them or when the physician believed that e-cigarettes were safer than conventional cigarettes.
Bascombe et al., 2016 [6]	Many participants mentioned that, in general, e-cigarette use does not come up in clinic visits very often; however, usually when it was mentioned, the conversation was about using them to quit smoking traditional cigarettes.
Cho et al., 2022 [17]	No difference between countries in the prevalence of nicotine vaping products (NVP) discussions from 2016 to 2020 (P = 0.0770).<br><br>The prevalence of health professionals (HPs) who initiated NVP discussion increased in England between 2016 and 2020 (53.3–72.8%; P = 0.0444) but did not significantly change in other countries (Australia: 53.0–39.9%, P = 0.9480; Canada: 39.9–49.9%, P = 0.4908; United States: 63.5–45.3%, P = 0.3793).<br><br>When smoking abstinence only was considered, compared to baseline exclusive daily smokers who did not discuss NVPs with HPs and did not discuss other cessation methods, those whose HPs recommended NVPs were not more likely to become quitters from smoking cigarettes regardless of whether they did or did not take up NVPs at follow-up (aRR = 0.93, 95% CI = 0.40, 2.14, P > 0.05).
Craig et al., 2022 [19]	Of the providers who discuss vaping in clinical settings, the majority (63%, 66/104) reported that they initiate discussions about vaping with their patients.<br><br>Providers reported that certain aspects of a patient's demographics and general appearance, such as an odour of tobacco or visualization of a patient's vaping unit, influence whether they ask the patient about electronic nicotine delivery systems (ENDS) usage.<br><br>About half (45%, 17/104) of providers denied that their patients have ever inquired about vaping, while 13% stated they frequently fielded questions about ENDS. However, only 18% (19/104) of providers felt "very comfortable" answering these questions.

	When broken down by question subject, such as side effects prevalence of ENDS use, the majority of providers demonstrated that they were most comfortable with explaining known side effects and pros/cons of vaping compared to traditional cigarette use.
Doescher et al., 2018 [22]	Nearly two thirds (62.0%) of the sample agreed with the statement that they felt comfortable talking with their primary care physician (PCP) about e-cigarettes.<br><br>Roughly one quarter (24.7%) wanted their PCP to talk with them about these products, although the percentage of patients who wanted their PCP to talk with them about e-cigarettes increased to nearly two thirds (62.0%) among recent e-cigarette users (P = 0.001).
El-Shahawy et al., 2016 [25]	Primary care physicians report that e-cigarette discussions are becoming commonplace in practice with patients initiating the discussions and seeking physician guidance regarding e-cigarette use.
Gallegos-Carrillo et al., 2020 [30]	Among smokers and vapers who had a health professional (HP) consultation during the last 4 months, 33.7% (n = 362) discussed e-cigarettes with their HP.<br><br>Dual users were significantly more likely to discuss with their HP about e-cigarettes (adjusted odds ratio (AOR) = 5.1; 95% C.I. 3.7, 7.2) than exclusive combustible cigarette smokers. Respondents who had an attempt to quit smoking in the last 4 months were also more likely to discuss e-cigarettes (AOR = 1.9; 95% C.I. 1.4, 2.7). In addition, those who reported their HP counselling them to quit smoking during the consultation were more likely to discuss e-cigarettes (AOR = 3.34; 95% C.I. 2.4, 4.6).<br><br>Among those who discussed e-cigarettes with their HP (n = 362), 46% reported that the HP brought up the topic. In adjusted models, the only statistically significant correlate was being a dual user (AOR = 1.74; 95% C.I. 1.05, 2.9).<br><br>Among respondents who talked about e-cigarettes with their health professional, 53.3% reported that the discussion led them to use an e-cigarette in a subsequent quit attempt, though 32.4% reported that the discussion persuaded them to have a quit attempt without using e-cigarettes in that attempt, while 14.3% indicated that their discussion carried out with the HP did not lead them to make a quit attempt.<br><br>Dual users were more likely than exclusive smokers to be persuaded to use e-cigarettes in their quit attempt (AOR = 2.6; 95% C.I. 1.5, 4.5).

	Respondents reporting that they plan to quit during the next six months (AOR = 1.7; 95% C.I. 1, 2.8) and whose HP advised them to quit smoking at all were more likely to be persuaded by their HP to use e-cigarettes in their quit attempt (AOR = 2.0; 95% C.I. 1.2, 3.4).
Gorukanti et al., 2022 [32]	10–17 year-old patients: Clinicians counselled 20.0% of patients on e-cigarette use. 18–26 year-old patients: Clinicians estimated counselling 30.0% of patients on e-cigarette use.
Gorzkowski et al., 2016 [33]	Clinical encounters involving e-cigarettes were rare: 65% of paediatricians had never discussed them; 35% stated it had happed at least once. Most expected these encounters to increase. Among those reporting clinical encounters, 85% said the user was a parent, and 92% reported that a parent/patient had broached the topic.
Gravely et al., 2019 [35]	Among the 6,615 smokers who visited a health professional (HP) in the last year, 6.8% reported discussing nicotine vaping products (NVPs) with an HP and 2.1% of smokers were encouraged to use an NVP (36.1% of those who had a discussion).<br><br>Compared with Australia (4.3%), discussing NVPs with an HP was more likely in the United States [8.8%, odds ratio (OR) = 2.15, 95% confidence interval (CI) = 1.41–3.29] and Canada (7.8%, OR = 1.87, 95% CI = 1.26–2.78). Smokers in Australia were less likely to discuss NVPs than smokers in England (6.2%), although this was not statistically significant (OR = 1.47, 95% CI = 0.98–2.20).
Heinly et al., 2023 [37]	Respondents report “advising” parents (5% vs 52%, P < .001) and “assisting” parents in quitting (2% vs 28%, P < .001) significantly less for e-cigarette users than for combustible tobacco users.
Hurst et al., 2018 [40]	Physicians who participated in occasional counselling about electronic nicotine delivery systems (ENDS) reported that nearly all their patients had independently experimented with different ENDS products before consulting with them for medical approval. Physicians noted that many patients who reported ENDS use were continuing to smoke combustible cigarettes (although in some cases reducing the number) and expressed reluctance to halt this dual use.
Jankowski et al, 2019 [44]	Physicians trained in a nonsurgical specialty used minimal intervention on smoking cessation more often with patients who smoke cigarettes (OR = 2.7; p < 0.001) or use e-cigarette (OR = 1.9; p < 0.05), compared to those trained in surgical specialties. Specialists more often (OR = 2.5; p < 0.05) referred e-cigarette users to smoking cessation clinics than physicians in training. Physicians who had never tried cigarettes more often referred smokers or e-cigarette users to smoking cessation clinics, compared to physicians who had tried at least one cigarette (OR = 2.3; p < 0.05; OR = 3.1; p < 0.01, respectively).
Kanchustambham et al., 2017 [46]	18% of physicians would advise e-cigarettes as nicotine-replacement therapy if asked by patients.
Kollath-Cattano et al., 2016 [49]	Of all participants who visited a physician in the previous 12 months, 15% reported talking to their physician about e-cigarettes. Compared with smokers who never used e-cigarettes, current e-cigarette users were more likely to talk with

	their physician about e-cigarettes (nondaily users: odds ratio [OR] = 2.70; daily users: OR = 4.29). Current daily e-cigarette users were also more likely to receive advice to use e-cigarettes for cessation (OR = 9.40) compared with those who never used e-cigarettes. Smokers who were male or who had recently attempted to quit were more likely than their counterparts to talk to a physician about e-cigarettes.
Kollath-Cattano et al., 2019 [50]	For most physicians, when the topic of e-cigarettes did emerge, it was initiated by the patient, mostly to say they were using e-cigarettes to quit smoking. One physician noted that "people will usually ask me before they do something," so most perceived their conversations with smokers to have occurred before they initiated e-cigarette use.
Mughal et al., 2018 [60]	Nearly all respondents reported giving cessation advice to cigarette users during consultations (98.1%), and this incrementally and significantly declined to 33.7% for e-cigarettes, 16.6% for waterpipe tobacco smoking, and 11.8% for smokeless tobacco ($P < 0.001$ for each product compared to cigarettes).
Pepper et al., 2015 [69]	18% of physicians often or always counselled about e-cigarettes vs. 79% for cigarettes ($p < .001$). Family medicine physicians also had higher odds of engaging in prevention of e-cigarette use than pediatricians (26% vs. 18%, adjusted OR 1.57, 95% CI 1.10, 2.25).
Peterson et al., 2018 [71]	Providers emphasized the medical uncertainty about the health effects of vaping as a means of influencing how patients perceive the potential risks of e-cigarettes. Some providers navigated conversations about uncertainty by linking the medical community's lack of knowledge about e-cigarette safety with the historical timeline of conventional cigarette knowledge acquisition. Providers reported success using this strategy, as their patients were aware of how knowledge of tobacco cigarette toxicity has evolved over the last century.<br><br>While discussing uncertainty about e-cigarettes, providers also expressed how it was important for them to be honest about the lack of medical consensus. Providers also used motivational interviewing, which focuses on understanding patients' intrinsic motivations to elicit behaviour change. Providers found success with giving patients specific external resources to complement in-person counselling, particularly for tech-savvy adolescents.<br><br>Providers found it difficult to discuss e-cigarettes in an already rushed visit. Providers also expressed confusion about the vaping-related slang used by adolescents. This prevented them from modifying their rhetoric to use age-appropriate language, as they commonly do with other risky behaviours (e.g. 'pot' for marijuana, 'sleeping together' for sex).
Sherratt et al., 2016 [76]	A large proportion of practitioners had been asked about e-cigarettes by patients who were ever-smokers within the past year; only 18.7 % (n = 25) of practitioners reported no patients having asked about e-cigarettes within the past year, whilst a small number reported more than three quarters of patients having asked about e-cigarettes within the past year (n = 4, 3.0 %).

	The results also demonstrated inconsistencies regarding the content of the advice provided to patients by practitioners. Some practitioners provided no advice or suggested that they had inadequate knowledge to advise patients (n = 12, 6.3 %).
Simoneau et al., 2017 [78]	Ninety-three percent of clinicians opined that e-cigarettes were equally or more dangerous than cigarettes but 34% never counselled youth about the dangers of e-cigarettes. More than half (65%) of the clinicians counselled adolescents who smoked cigarettes about the dangers of e-cigarettes at least annually, but only 57% counselled non-smoking adolescents about the dangers of e-cigarettes at least annually.
Singh et al., 2017 [79]	Of the 35 physicians interviewed, only 2 (both OB/GYNs) reported never having discussed e-cigarettes with patients. Among the physicians who reported conversations with patients about e-cigarettes, the conversations were mainly prompted by the patient informing the physician that he/she had tried or wanted to try e-cigarettes to quit smoking, and he/she sought physician advice on e-cigarette safety and efficacy as a cessation device. In the few instances where a physician initiated the conversation, the exchange was the result of seeing a patient physically holding an e-cigarette or identifying themselves as a user of e-cigarettes.<br><br>When asked by patients about the safety and efficacy of e-cigarettes, physicians from all specialties were open with patients about the lack of information on the long-term health effects of e-cigarettes and that they cannot definitively say whether it is a good or bad product for smoking cessation based on the evidence available.
Singh et al., 2024 [80]	Only a third of GPs (34%) discussed e-cigarettes with a child or adolescent attending their clinics for other health concerns. In response to the question regarding the percentage of the population approaching the GPs to discuss e-cigarettes, about half of the GPs (40%) reported that an adolescent or a parent has never approached them to discuss e-cigarettes.
Stepney et al., 2019 [83]	Although interviewees' confidence levels varied when having conversations with patients (some said they were 'reasonably confident' in advising patients about e-cigarettes, whereas others said they were 'unsure' or 'apprehensive'), many took the approach of having an 'honest conversation' with patients regarding their own knowledge, the long-term effects, and unknowns.
Tildy et al., 2023 [87]	Among respondents who reported visiting a health professional (HP) in the last 18 months, 6.1% (n = 859) reported that their HP discussed nicotine vaping products with them.
Van Gucht et al., 2016 [88]	About half of the respondents support patients who spontaneously tell them that they want to start using e-cigarettes. GPs were significantly more supportive than tobacco counsellors (68% vs. 41%; $p < .05$).

Westmaas et al., 2023 [89]	Greater frequency of discussing patients' use of e-cigarettes was associated with greater comfort treating patients' smoking (p ≤.001), with greater perceived knowledge of e-cigarettes (p ≤.0001), and was more frequently engaged in by nurse practitioners (p ≤ .001) and physician assistants (p ≤ .03) compared to oncologists.
Znyk et al., 2024 [94]	Nurses who were knowledgeable and skilled in e-cigarette use (OR=1.79; p < 0.05), who measured weight, height, and BMI (OR=2.54; p < 0.001), and who smoked tobacco (OR=2.44; p < 0.01) and who had a family history of coronary artery disease (OR=1.65; p < 0.05) were more likely to provide minimal anti-smoking intervention to e-cigarette users.
**Prescribing**	
Balaraman et al., 2022 [4]	Participants do not actively recommend e-cigarettes (EC) to their patients. However, they informed that they will not discourage interested patients from trying EC as a smoking cessation aid, particularly those who failed to quit with other smoking cessation methods. The physicians were more likely to recommend EC when their patients asked about them or when the physician believed that EC was safer than conventional cigarettes.
Bascombe et al., 2016 [6]	There were many instances where participants expressed their interest in finding out if e-cigarettes can be used for smoking cessation. Some participants had already recommended using them instead of traditional cigarettes for some of their patients.
Bostan et al., 2022 [9]	98.8% to 99.0% of physicians in the study have recommended e-cigarettes to their patients before (closed and open tank e-cigarettes, respectively).
Brett et al., 2020 [10]	29% of clinicians would not recommend e-cigarettes to patients with cancer who continue to smoke. Recommendations of e-cigarettes associated with smoking cessation knowledge, e-cigarette knowledge, engagement with patients regarding smoking cessation, belief in the effectiveness of e-cigarettes, belief in sufficient evidence on e-cigarettes, and clinician conform with discussion e-cigarettes with patients.
Busher et al., 2024 [14]	Most respondents (78.1%) never recommend e-cigarettes to patients.
Cho et al., 2022 [17]	In 2020, a low percentage of smokers who discussed nicotine vaping products (NVPs) with a health professional (HP) reported that their HPs recommended they use NVPs in the United States (14.7%), Australia (20.2%), Canada (25.7%), with a higher percentage in England (55.7%) where clinical guidelines for smoking cessation include NVPs.<br><br>Compared with 12.0% of smokers who reported no discussion, 37.0% of those whose HPs recommended NVPs transitioned to vaping at follow-up. Transition to quitting was 9.6% with HPs' recommendation of NVPs versus 13.5% without discussion, a nonsignificant difference.
Delnevo et al., 2022 [20]	21.7% reported ever recommending e-cigarettes to a patient (9.8% in the past 30 days).

Egnot et al., 2017 [24]	25 (17.7%) oh resident physicians reported that they have been recommending electronic cigarettes to patients for the past 6 months or longer (action/maintenance). Residents were more likely to recommend e-cigarettes if they reported having a strong knowledge base, had encountered print advertisement or read a peer reviewed article, reported current smoking and were less likely to report that abstinence should be the primary goal of smoking cessation treatment.<br><br>Recommendation of electronic cigarettes was associated with residents ≥PGY-3 (OR=3.68, 95% CI 1.20 to 11.29), peer-reviewed article exposure (OR=6.65, 95% CI 1.56 to 28.38) and the view that addictive potential is less than with traditional cigarettes (OR=5.05, 95% CI 1.48 to 17.24).
El-Shahawy et al., 2016 [25]	Six of 13 primary care physicians' (PCPs') reported having previously recommended e-cigarette use to at least one of their patients.
Erku et al., 2019 [27]	It was common for pharmacy staff to identify a need to educate or counsel customers and to provide recommendations for or against use (33%).
Gallegos-Carrillo et al., 2020 [30]	Almost half (46%) of smokers who discussed e-cigarettes indicated that their health professional (HP) recommended their use, 23.5% reported being advised against their use and 29.6% indicated their HP did not express an opinion either for or against e-cigarettes use. Smokers who reported that their HP had counselled them to quit smoking were more likely to report that their HP recommended them to use e-cigarettes (AOR 1.7; 95% C.I. 1.0, 2.7).
Gravely et al., 2019 [35]	Among those who had discussed smoking cessation (n = 724), 37.8% received advice to use nicotine vaping products (NVPs), 20.9% of smokers were advised against NVP use and 41.3% of health professionals (HPs) remained neutral.<br><br>Overall, the prevalence of HPs recommending NVPs was three times more likely in the United States than in Australia (OR = 3.07, 95% CI = 1.45–6.47), and twice as likely in Canada (OR = 2.28, 95% CI = 1.06–4.87) than in Australia. Australia and England did not differ (OR = 1.76, 95% CI = 0.83–3.74).
Jackson et al., 2021 [43]	3.7% of 11,588 smokers reported having been recommended to use an e-cigarette.
Kanchustambham et al., 2017 [46]	21 (18.2%) participants had recommended patients to use e-cigarettes.
Kandra et al., 2014 [47]	35.2% of physicians recommended e-cigarettes to their patients.
Kollath-Cattano et al., 2016 [49]	Among those asked whether their physician recommend e-cigarettes for smoking cessation (n = 257), 61% responded affirmatively.

Kollath-Cattano et al., 2019 [50]	Both physicians who included e-cigarettes as one of the viable strategies for smoking cessation did not recommend them in lieu of best-practice recommendations. Two physicians who were against e-cigarettes, one of whom reported they had never seen patients successfully use them to quit, stated that they would recommend cessation of e-cigarette use to current users. Conversely, the two physicians who regularly include e-cigarettes as an option for smoking cessation reported frequent smoking cessation success with patients who used e-cigarettes. The remaining physicians were more ambivalent about e-cigarettes.
Koo et al., 2022 [51]	7.35% (n =10) recommended e-cigarettes to be used as one of the smoking cessation tools.
Koprivnikar et al., 2020 [52]	While a minority of healthcare professional participants (12.7%) do or would recommend electronic cigarettes for smoking cessation or reduction in general, a higher proportion of (33.1%) would recommend electronic cigarettes to specific groups of patients.
Mohammad et al., 2023 [57]	The statement *I advise my patient to use e-cigs as a replacement for c-cigs* had a mean rating of 2.2 (SD ± 1), based on a five-point Likert scale from strongly disagree (1) to strongly agree (5). This score was significantly higher ($p < 0.001$) for healthcare professionals who were ever-users of e-cigarettes.
Moysidou et al., 2016 [59]	Only 14.5% knew about the pending European regulation on e-cigarettes, but 33.2% had recommended them to smokers in the past. Still, more than 40% would not recommend electronic cigarettes to smokers unwilling or unable to quit smoking with currently approved medications.
Nickels et al., 2017 [64]	38% of physicians have at some point recommended electronic cigarettes to their patients that smoke, with 12% reporting recommending them at least 25% of the time.
Northrup et al., 2017 [65]	4.4% (n = 17) of physicians prescribed/recommended e-cigarettes to quit for pregnant smokers who wish to quit.
Ofei-Dodoo et al., 2017 [66]	Of the 117 respondents, 18% indicated they do recommend e-cigarettes for smoking cessation.
Ofei-Dodoo et al., 2020 [67]	Of the 247 respondents, 13.6% reported they recommended e-cigarettes for tobacco use cessation. Just over 86% of the 247 family physicians did not recommend e-cigarettes for tobacco use cessation.
Salloum et al., 2021 [74]	Primary care physicians (PCPs) recommended e-cigarettes in 27% of patient profiles they evaluated. Less than 10% of PCPs indicated they recommend e-cigarettes for cessation often or always, while almost half stated they never recommended e-cigarettes for cessation. Most PCPs stated they encourage patients to try other cessation methods before e-cigarettes often or always.
Selamoglu et al., 2024 [75]	Some participants described how they were adamant they would not recommend e-cigarettes. Some participants who had tried established treatments to help their patients cease smoking without success, were willing to recommend e-

	cigarettes as a “third line option” and as a “last resort” to patients that have “tried varenicline, bupropion, NRTs, counselling, behavioural support and hasn’t worked.”
Sherratt et al., 2016 [76]	Some practitioners provided no advice or suggested that they had inadequate knowledge to advise patients (n = 12, 6.3 %).
Singh et al., 2017 [79]	While physicians informed patients that there is a lack of data on the safety and efficacy of e-cigarettes and did not actively recommend them, they did not discourage patients from using them.
Singh et al., 2024 [80]	When questioned about the practice of GPs using e-cigarettes as a smoking cessation tool, most GPs (79%) had never recommended e-cigarettes to adolescents who smoked conventional cigarettes, although the rest had either rarely or occasionally made that recommendation.
Smith et al., 2019 [81]	Judgements regarding the suitability of using electronic cigarettes (ECs) for tobacco harm reduction (THR) within a clinical setting were sometimes dependent on the treatment context and patients' individual circumstances. For instance, one mental health professional (MHP) expressed how decisions regarding whether to allow patients to use ECs, or even conventional cigarettes, depended upon the patients' stage of treatment.<br><br>Patient choice was taken into consideration by some MHPs when determining whether ECs would be a helpful THR product for patients within their care.<br><br>Even among MHPs who were sceptical of ECs, a minority spoke of accounts whereby they had encouraged EC use by patients. Although such accounts were rare and predominantly in the context of inpatient settings, some MHPs expressed how they would encourage patients who are already using ECs to take them on escorted leave, in attempt to dissuade them from purchasing tobacco which would inflict financial strain on the patient.<br><br>One MHP spoke about how, despite previously raising concerns regarding ECs, she allows ECs to be used by patients in their rooms. Moreover, this MHP elaborated on how, despite being ill-informed regarding ECs, she had read the smoke-free policy and believed they serve to be a popular alternative source of nicotine for patients who could not smoke.
Steinberg et al., 2015 [82]	Almost a third (30%) of physicians reported that they have recommended e-cigarettes as a smoking cessation tool.
Tanriover et al., 2022 [86]	Of the family physicians, 6.6% have stated that they have recommended e-cigarettes to their patients for smoking cessation with the strategy of harm reduction.

Tildy et al., 2023 [87]	Among respondents who reported visiting a heath professional (HP) in the last 18 months and reported that the HP discussed nicotine vaping products (NVPs) with them, one-third (33.5%, n = 288) reported receiving a positive recommendation from their HP to use NVPs.

References

1. Agbonlahor O, Mattingly DT, Hart JL, Rai J, McLeish AC, Walker KL. Differences in health care provider advice on abstaining from tobacco use: findings from the 2020 National Youth Tobacco Survey. Addict Behav. 2023;144:107726.
2. Akhter S, Ghazal S, Rizvi N, Aziz HW, Warraich UA. Knowledge, attitude and perception regarding e-cigarette among postgraduate medical trainees in Pakistan. Rawal Med J. 2023;48(2):352-6.
3. Albury C, Barnes R, Ferrey A, Coleman T, Gilbert H, Naughton F et al. The old and familiar meets the new and unknown: patient and clinician perceptions on e-cigarettes for smoking reduction in UK general practice, a qualitative interview study. Addiction. 2022;117(5):1427-37.
4. Balaraman AK, Aziz Ur R, Xuan YW, Keshavarzi F, Farrukh MJ. Smoking cessation practitioners views towards safety and effectiveness of electronic cigarettes in Klang Valley, Malaysia: a qualitative study approach. Curr Trends Biotechnol Pharm. 2022;16(1):77-88.
5. Baldassarri SR, Chupp GL, Leone FT, Warren GW, Toll BA. Practice patterns and perceptions of chest health care providers on electronic cigarette use: an in-depth discussion and report of survey results. J Smok Cessat. 2018;13(2):72-7.
6. Bascombe TM, Scott KN, Ballard D, Smith SA, Thompson W, Berg CJ. Primary healthcare provider knowledge, beliefs and clinic-based practices regarding alternative tobacco products and marijuana: a qualitative study. Health Educ Res. 2016;31(3):375-83.
7. Bell SK, Mena G, Dean J, Boyd M, Gilks C, Gartner C. Vaporised nicotine and tobacco harm reduction for addressing smoking among people living with HIV: a cross-sectional survey of Australian HIV health practitioners' attitudes. Drug Alcohol Depend. 2017;177:67-70.
8. Boakye E, Osuji N, Erhabor J, Obisesan O, Osei AD, El Shahawy O et al. Healthcare provider screening for tobacco product and electronic cigarette use among youth in the United States. J Adolesc Health. 2023;72(5):819-22.
9. Bostan P, Gorek Dilektasli A. The knowledge and attitude about new generation tobacco products among physicians. Turk Thorac J. 2022;23(6):369-75.
10. Brett J, Davies EL, Matley F, Aveyard P, Wells M, Foxcroft D et al. Electronic cigarettes as a smoking cessation aid for patients with cancer: beliefs and behaviours of clinicians in the UK. BMJ Open. 2020;10(11):e037637.
11. Broadfield A, Ayre H, Ahmadi K. Electronic cigarettes for smoking cessation in pregnancy: a nation–wide mixed methods study. Br J Midwifery. 2023;31(3):133-41.
12. Brown-Johnson CG, Burbank A, Daza EJ, Wassmann A, Chieng A, Rutledge GW et al. Online patient-provider e-cigarette consultations: perceptions of safety and harm. Am J Prev Med. 2016;51(6):882-9.
13. Bruno FP, Degani-Costa LH, Kandipudi KLP, Gushken F, Szlejf C, Tokeshi AB et al. Medical trainees' knowledge and attitudes towards electronic cigarettes and hookah: a multinational survey study. Respir Care. 2024;69(3):306-16.
14. Busher A, McAvoy H, Cox DW, Kavanagh PM. Knowledge, attitudes and practices of Irish doctors regarding stop smoking care and electronic cigarettes. Ir J Med Sci. 2024;193(2):783-90.
15. Caponnetto P, Maglia M, Mangione M, Vergopia C, Prezzavento GC, Polosa R et al. Smoking addiction in patients with schizophrenia spectrum disorders and its perception and intervention in healthcare personnel assigned to psycho-rehabilitation programs: a qualitative research. Healthcare (Basel). 2022;10(11):2275.
16. Chinwong S, Penthinapong T, Tengcharoenphongthorn P, Pingkaew S, Siriwattana K, Phrommintikul A et al. Electronic cigarettes and tobacco product cessation: a survey of

healthcare providers' opinions on safety and recommendation. Healthcare (Basel). 2024;12(14):1410.
17. Cho YJ, Thrasher JF, Gravely S, Alberg A, Borland R, Yong HH et al. Adult smokers' discussions about vaping with health professionals and subsequent behavior change: a cohort study. Addiction. 2022;117(11):2933-42.
18. Clegg H, Howle F, Groom K, Moore R, Hryhorskyj L, Grundy S et al. Understanding the enablers and barriers to implementing smoke-free NHS sites across acute care trusts in Greater Manchester: results of a hospital staff survey. Future Healthc J. 2021;8(3):e676-82.
19. Craig M, Tamirisa A, Madhavan R, Jochum E, Cohen A, Zoorob D. Vaping in pregnancy: a geographically focused assessment of OBGYN provider knowledge regarding the safety and usage of electronic nicotine delivery systems. Clin Exp Obstet Gynecol. 2022;49(2):31.
20. Delnevo CD, Jeong M, Teotia A, Bover Manderski MM, Singh B, Hrywna M et al. Communication between US physicians and patients regarding electronic cigarette use. JAMA Netw Open. 2022;5(4):e226692.
21. Deng R, Yang C, Yuan Y, Liang L, Yang X, Wang X et al. A cross-sectional survey of medical and other groups' awareness, perceptions, and willingness to use e-cigarettes during the COVID-19 pandemic. Front Public Health. 2023;11:1323804.
22. Doescher MP, Wu M, Rainwater E, Khan AS, Rhoades DA. Patient perspectives on discussions of electronic cigarettes in primary care. J Am Board Fam Med. 2018;31(1):73-82.
23. Dwedar I, Ruby D, Mostafa A. A survey exploring knowledge and beliefs about electronic cigarettes between health care providers and the general population in Egypt. Int J Chron Obstruct Pulmon Dis. 2019;14:1943-50.
24. Egnot E, Jordan K, Elliott JO. Associations with resident physicians' early adoption of electronic cigarettes for smoking cessation. Postgrad Med J. 2017;93(1100):319-25.
25. El-Shahawy O, Brown R, Elston Lafata J. Primary care physicians' beliefs and practices regarding e-cigarette use by patients who smoke: a qualitative assessment. Int J Environ Res Public Health. 2016;13(5):445.
26. England LJ, Anderson BL, Tong VT, Mahoney J, Coleman-Cowger VH, Melstrom P et al. Screening practices and attitudes of obstetricians-gynecologists toward new and emerging tobacco products. Am J Obstet Gynecol. 2014;211(6):695.e1-7.
27. Erku DA, Gartner CE, Do JT, Morphett K, Steadman KJ. Electronic nicotine delivery systems (e-cigarettes) as a smoking cessation aid: a survey among pharmacy staff in Queensland, Australia. Addict Behav. 2019;91:227-33.
28. Feng Y, Wang F, Abdullah AS, Wang X, Wang J, Zheng P. Beliefs, attitudes, and confidence to deliver electronic cigarette counseling among 1023 Chinese physicians in 2018. Int J Environ Res Public Health. 2019;16(17):3175.
29. Ferrara P, Shantikumar S, Cabral Verissimo V, Ruiz-Montero R, Masuet-Aumatell C, Ramon-Torrell JM. Knowledge about e-cigarettes and tobacco harm reduction among public health residents in Europe. Int J Environ Res Public Health. 2019;16(12):2071.
30. Gallegos-Carrillo K, Barrientos-Gutierrez I, Arillo-Santillan E, Zavala-Arciniega L, Cho YJ, Thrasher JF. Health professionals' counseling about electronic cigarettes for smokers and vapers in a country that bans the sales and marketing of electronic cigarettes. Int J Environ Res Public Health. 2020;17(2):442.
31. Geletko KW, Myers K, Brownstein N, Jameson B, Lopez D, Sharpe A et al. Medical residents' and practicing physicians' e-cigarette knowledge and patient screening activities: Do they differ? Health Serv Res Manag Epidemiol. 2016;3:2333392816678493.
32. Gorukanti AL, Kimminau KS, Tindle HA, Klein JD, Gorzkowski J, Kaseeska K et al. Cross-sectional online survey of clinicians' knowledge, attitudes and challenges to screening

and counselling adolescents and young adults for substance use. BMJ Open. 2022;12(11):e059019.
33. Gorzkowski JA, Whitmore RM, Kaseeska KR, Brishke JK, Klein JD. Pediatrician knowledge, attitudes, and practice related to electronic cigarettes. J Adolesc Health. 2016;59(1):81-6.
34. Gould GS, Zeev YB, Tywman L, Oldmeadow C, Chiu S, Clarke M et al. Do clinicians ask pregnant women about exposures to tobacco and cannabis smoking, second-hand-smoke and e-cigarettes? An Australian national cross-sectional survey. Int J Environ Res Public Health. 2017;14(12):1585.
35. Gravely S, Thrasher JF, Cummings KM, Ouimet J, McNeill A, Meng G et al. Discussions between health professionals and smokers about nicotine vaping products: results from the 2016 ITC Four Country Smoking and Vaping Survey. Addiction. 2019;114 Suppl 1(Suppl 1):71-85.
36. Haber LA, Ortiz GM. Clearing the air: inpatient providers' knowledge, perspectives, and experience with electronic cigarettes. J Hosp Med. 2014;9(12):805-7.
37. Heinly A, Baird J, Riese A. Screening and counseling practices for parental combustible versus electronic cigarette use in pediatric primary care: a pilot study. Clin Pediatr (Phila). 2023;62(10):1169-1175.
38. Huang P, Zheng W, Shi Y, Shi L, Wu W, Lin X et al. Beliefs and perceptions of electronic cigarettes among medical staff in respiratory departments of Fujian Province, China, in 2021. Tob Induc Dis. 2022;20(December):111.
39. Hunter A, Yargawa J, Notley C, Ussher M, Bobak A, Murray RL et al. Healthcare professionals' beliefs, attitudes, knowledge, and behavior around vaping in pregnancy and postpartum: a qualitative study. Nicotine Tob Res. 2021;23(3):471-478.
40. Hurst S, Conway M. Exploring physician attitudes regarding electronic documentation of e-cigarette use: a qualitative study. Tob Use Insights. 2018;11:1179173X18782879.
41. Hwang J, Lee C, Mastrolonardo E, Frasso R. Where there's smoke, there's fire: what current and future providers do and do not know about electronic cigarettes. BMC Public Health. 2020;20(1):1145.
42. Isett KR, Rosenblum S, Barna JA, Hicks D, Gilbert GH, Melkers J. Missed opportunities for detecting alternative nicotine product use in youth: data from the national dental practice-based research network. J Adolesc Health. 2018;63(5):587-593.
43. Jackson SE, Garnett C, Brown J. Prevalence and correlates of receipt by smokers of general practitioner advice on smoking cessation in England: a cross-sectional survey of adults. Addiction. 2021;116(2):358-372.
44. Jankowski M, Kaleta D, Zgliczynski WS, Grudziaz-Sekowska J, Wrzesniewska-Wal I, Gujski M et al. Cigarette and e-cigarette use and smoking cessation practices among physicians in Poland. Int J Environ Res Public Health. 2019;16(19).
45. Jongebloed H, Cole E, Dean E, Ugalde A. The role of general practice nurses in supporting people to quit smoking: a qualitative study. PLoS One. 2024;19(7):e0306555.
46. Kanchustambham V, Saladi S, Rodrigues J, Fernandes H, Patolia S, Santosh S. The knowledge, concerns and healthcare practices among physicians regarding electronic cigarettes. J Community Hosp Intern Med Perspect. 2017;7(3):144-150.
47. Kandra KL, Ranney LM, Lee JG, Goldstein AO. Physicians' attitudes and use of e-cigarettes as cessation devices, North Carolina, 2013. PLoS One. 2014;9(7):e103462.
48. Khalaf AT, Doustjalali SR, Sabet NS, Lin WM, Linn NH, Dar HT et al. Knowledge and perception of nicotine, nicotine replacement therapy and ecigarettes among health care workers in Chengdu. J Pharm Negat Results. 2023;14(2).
49. Kollath-Cattano C, Thrasher JF, Osman A, Andrews JO, Strayer SM. Physician advice for e-cigarette use. J Am Board Fam Med. 2016;29(6):741-747.

50. Kollath-Cattano C, Dorman T, Albano AW, Jr., Jindal M, Strayer SM, Thrasher JF. E-cigarettes and the clinical encounter: physician perspectives on e-cigarette safety, effectiveness, and patient educational needs. J Eval Clin Pract. 2019;25(5):761-768.
51. Koo EY, Kozak KJ, Achim V, Wenig BL. E-cigarette use and tobacco harm reduction: pilot survey study evaluating perspectives of head and neck surgeons. Head Neck. 2022;44(2):582-585.
52. Koprivnikar H, Zupanic T, Farkas JL. Beliefs and practices regarding electronic cigarettes in smoking cessation among healthcare professionals in Slovenia. Tob Prev Cessat. 2020;6:3.
53. Luxton NA, Shih P, Rahman MA. Electronic cigarettes and smoking cessation in the perioperative period of cardiothoracic surgery: views of Australian clinicians. Int J Environ Res Public Health. 2018;15(11).
54. Marques Gomes AC, Nabhani-Gebara S, Kayyali R, Buonocore F, Calabrese G. Survey of community pharmacists' perception of electronic cigarettes in London. BMJ Open. 2016;6(11):e013214.
55. McConaha J, Grabigel A, DiLucente D, Lunney PD. Electronic cigarettes: the perceptions of pharmacists and physicians. Pharmacotherapy. 2017;13(1):26-32.
56. McGee LU, Parker S, Bulsara S, Escobar B, Kline KN, Jibaja-Weiss ML et al. Adolescent electronic cigarette counselling: knowledge, attitudes and perceived barriers among clinical staff in a primary care setting. J Eval Clin Pract. 2021;27(6):1398-402.
57. Mohammad H, Nassrawin S, Alhyasat A, AlMuhaissen S. Physicians, pharmacists, and dentists’ knowledge and attitudes toward the use of electronic cigarettes. Clin Epidemiol Glob Health. 2023;24:101443.
58. Morphett K, Holland A, Ward S, Steadman KJ, Zwar NA, Gartner C. Evaluating the implementation of a prescription only regulatory model for nicotine vaping products: a qualitative study on the experiences and views of healthcare professionals. Int J Drug Policy. 2024;125:104353.
59. Moysidou A, Farsalinos KE, Voudris V, Merakou K, Kourea K, Barbouni A. Knowledge and perceptions about nicotine, nicotine replacement therapies and electronic cigarettes among healthcare professionals in Greece. Int J Environ Res Public Health. 2016;13(5):514.
60. Mughal F, Rashid A, Jawad M. Tobacco and electronic cigarette products: awareness, cessation attitudes, and behaviours among general practitioners. Prim Health Care Res Dev. 2018;19(6):605-9.
61. Mungia R, Case K, Valerio MA, Mendoza M, Taverna M, de la Rosa EM et al. Development of an e-cigarettes education and cessation program: A south Texas oral health network study. Health Promot Pract. 2021;22(1):18-20.
62. Mungia R, Mexquitic M, Case K, Atique M, Jones B, MacCarthy D et al. Implementation of a youth and young adult e-cigarette cessation program within a dental clinic setting : a SToHN feasibility study. Tex Dent J. 2022;139(9):542-54.
63. Naylor H, Howie A, Every-Palmer S. Vaping in mental health inpatient units: a qualitative study of consumer and staff views on electronic nicotine delivery systems. Australas Psychiatry. 2024;32(3):230-4.
64. Nickels AS, Warner DO, Jenkins SM, Tilburt J, Hays JT. Beliefs, practices, and self-efficacy of US physicians regarding smoking cessation and electronic cigarettes: a national survey. Nicotine Tob Res. 2017;19(2):197-207.
65. Northrup TF, Klawans MR, Villarreal YR, Abramovici A, Suter MA, Mastrobattista JM et al. Family physicians' perceived prevalence, safety, and screening for cigarettes, marijuana, and electronic-nicotine delivery systems (ENDS) use during pregnancy. J Am Board Fam Med. 2017;30(6):743-57.

66. Ofei-Dodoo S, Kellerman R, Nilsen K, Nutting R, Lewis D. Family physicians' perceptions of electronic cigarettes in tobacco use counseling. J Am Board Fam Med. 2017;30(4):448-59.
67. Ofei-Dodoo S, Wipperman J, Nutting R, Gilchrist K, Kellerman R. Changes in family physicians' perceptions of electronic cigarettes in tobacco use counseling between 2016 and 2019. Kans J Med. 2020;13:311-7.
68. Oliver AP, Bell LA, Agley J, Bixler K, Hulvershorn LA, Adams ZW. Examining the efficacy of project ECHO to improve clinicians' knowledge and preparedness to treat adolescent vaping. Clin Pediatr (Phila). 2022;61(12):869-8.
69. Pepper JK, Gilkey MB, Brewer NT. Physicians' counseling of adolescents regarding e-cigarette use. J Adolesc Health. 2015;57(6):580-6.
70. Pepper JK, McRee AL, Gilkey MB. Healthcare providers' beliefs and attitudes about electronic cigarettes and preventive counseling for adolescent patients. J Adolesc Health. 2014;54(6):678-83.
71. Peterson EB, Fisher CL, Zhao X. Pediatric primary healthcare providers' preferences, experiences and perceived barriers to discussing electronic cigarettes with adolescent patients. J Commun Healthc. 2018;11(4):245-51.
72. Rahman N, Sebar B, Sofija E. "It's the wild west out there": a qualitative study of the views and preparedness of health professionals in helping young adult e-cigarette users to quit. Nicotine Tob Res. 2024;26(11):1538-44.
73. Ridner SL, Keith RJ, Walker KL, Hart JL, Robertson SE. Primary care nurse practitioners' perceptions of the use of e-cigarettes. J Nurse Pract. 2017;13(6):e283-6.
74. Salloum RG, LeLaurin JH, Lee JH, Lafata JE, Williams M, Wang Y et al. Primary care physician perspectives on recommending e-cigarettes to smokers: a best-worst discrete choice experiment. J Gen Intern Med. 2021;36(11):3353-60.
75. Selamoglu M, Erbas B, Wilson H, Barton C. 'Why do we have to be the gatekeepers?' Australian general practitioners' knowledge, attitudes and prescribing intentions on e-cigarettes as a smoking cessation aid. BMC Prim Care. 2024;25(1):53.
76. Sherratt FC, Newson L, Field JK. Electronic cigarettes: a survey of perceived patient use and attitudes among members of the British thoracic oncology group. Respir Res. 2016;17(1):55.
77. Shin DW, Kim YI, Kim SJ, Kim JS, Chong S, Park YS et al. Lung cancer specialist physicians' attitudes towards e-cigarettes: a nationwide survey. PLoS One. 2017;12(2):e0172568.
78. Simoneau T, Hollenbach JP, Langton CR, Kuo CL, Cloutier MM. Smoking cessation and counseling: a mixed methods study of pediatricians and parents. PLoS One. 2021;16(2):e0246231.
79. Singh B, Hrywna M, Wackowski OA, Delnevo CD, Jane Lewis M, Steinberg MB. "Knowledge, recommendation, and beliefs of e-cigarettes among physicians involved in tobacco cessation: a qualitative study". Prev Med Rep. 2017;8:25-9.
80. Singh R, Burke M, Towns S, Rahman MA, Bittoun R, Shah S et al. Exploring general practitioners' knowledge, attitudes, and practices towards e-cigarette use/vaping in children and adolescents: a pilot cross-sectional study in sydney. Int J Environ Res Public Health. 2024;21(9):1215.
81. Smith CA, McNeill A, Kock L, Ahmed Z, Shahab L. Mental health professionals' perceptions, judgements and decision-making practices regarding the use of electronic cigarettes as a tobacco harm reduction intervention in mental healthcare: a qualitative focus group study. Addict Behav Rep. 2019;10:100184.
82. Steinberg MB, Giovenco DP, Delnevo CD. Patient-physician communication regarding electronic cigarettes. Prev Med Rep. 2015;2:96-8.
83. Stepney M, Aveyard P, Begh R. GPs' and nurses' perceptions of electronic cigarettes in England: a qualitative interview study. Br J Gen Pract. 2019;69(678):e8-14.

84. Stevens MWR, Cooper M, Cusack L, Ali RL, Holmwood C, Briley AL. Screening and early intervention for substance use during pregnancy: a retrospective case note review of antenatal care records. Drug Alcohol Rev. 2024;43(7):1817-28.
85. Talley B, Dube S, Chandora R, Nayak P, Eriksen MP. Addiction, cessation, & harm reduction: primary care provider knowledge & perceptions of electronic nicotine delivery system. Osteopath Fam Physician. 2017;9(2):10-6.
86. Tanriover O, Hidiroglu S, Ay P, Cook RL. Do family physicians perceive electronic cigarette use as a harm reduction strategy for smokers? A survey from Istanbul. Prim Health Care Res Dev. 2022;23:e15.
87. Tildy BE, McNeill A, East K, Gravely S, Fong GT, Cummings KM et al. Self-reported depression and anxiety and healthcare professional interactions regarding smoking cessation and nicotine vaping: findings from 2018 International Tobacco Control Four Country Smoking and Vaping (ITC 4CV) Survey. Tob Prev Cessat. 2023;9:26.
88. Van Gucht D, Baeyens F. Health professionals in Flanders perceive the potential health risks of vaping as lower than those of smoking but do not recommend using e-cigarettes to their smoking patients. Harm Reduct J. 2016;13(1):22.
89. Westmaas JL, Kates I, Makaroff L, Henson R. Barriers to helping patients quit smoking: lack of knowledge about cessation methods, e-cigarettes, and why nurse practitioners and physician assistants can help. Public Health Pract (Oxf). 2023;6:100409.
90. Zgliczynski WS, Jankowski M, Rostkowska O, Gujski M, Wierzba W, Pinkas J. Knowledge and beliefs of e-cigarettes among physicians in Poland. Med Sci Monit. 2019;25:6322-30.
91. Zhong Q, An K, Wu Z, Zhang H, Li S, Zhang L et al. Knowledge and awareness of nicotine, nicotine replacement therapy, and electronic cigarettes among general practitioners with a special interest in respiratory medicine in China. Front Med (Lausanne). 2023;10:1236453.
92. Zhou SS, Baptist AP. Electronic cigarettes: how confident and effective are allergists, pulmonologists, and primary care physicians in their practice behavior? Allergy Asthma Proc. 2020;41(3):192-7.
93. Zijlstra DN, Hoving C, Bolman C, Muris JWM, De Vries H. Do professional perspectives on evidence-based smoking cessation methods align? A Delphi study among researchers and healthcare professionals. Health Educ Res. 2022;36(4):434-5.
94. Znyk M, Kostrzewski S, Kaleta D. Nurse-led lifestyle counseling in Polish primary care: the effect of current health status and perceived barriers. Front Public Health. 2024;12:1301982.
